# From structure to function: tunable electrical and catalytic properties in rare Mo(vi)-thiophene-2-carboxylic acid hydrazone complexes obtained mechanochemically

**DOI:** 10.1039/d5ra07456h

**Published:** 2025-11-26

**Authors:** Luka Pavić, Filip Miočić, Marta Razum, Josipa Sarjanović, Jana Pisk

**Affiliations:** a Division of Materials Chemistry, Ruđer Bošković Institute 10000 Zagreb Croatia lpavic@irb.hr; b Department of Chemistry, Faculty of Science, University of Zagreb Horvatovac 102a 10000 Zagreb Croatia jana.pisk@chem.pmf.hr

## Abstract

This study focuses on the synthesis of molybdenum complexes coordinated with a thiophene-2-carboxylic acid hydrazone-type ligand (H_2_L), obtained *via* mechanochemical reaction condensation of 2-hydroxybenzaldehyde with thiophene-2-carboxylic acid hydrazide. The Mo complexes were prepared *via* a green mechanochemical route, [MoO_2_(L)(MeOH)] and [MoO_2_(L)]_*n*_, while the complex [MoO_2_(L)(H_2_O)] was obtained by classic solution-based synthetic pathway. All obtained compounds were characterized using attenuated total reflectance infrared spectroscopy (IR-ATR), elemental analysis (EA), and thermogravimetric analysis (TGA). The crystal and molecular structures of the ligand H_2_L and the complexes [MoO_2_(L)(MeOH)] and [MoO_2_(L)(H_2_O)]·(CH_3_)_2_CO were elucidated by means of single-crystal X-ray diffraction (SCXRD) analysis. Catalytic studies revealed that complex [MoO_2_(L)(MeOH)] efficiently promoted the oxidation of benzyl alcohol under mild conditions, employing hydrogen peroxide as a green oxidant. Solid-state impedance spectroscopy (ss-IS) confirmed the semiconducting behavior, with DC conductivities of ∼10^−12^ (Ω cm)^−1^ and activation energies of ∼60–63 kJ mol^−1^, consistent with electronic transport. Dielectric insights revealed frequency-dependent polarization processes dominated by Maxwell–Wagner interfacial effects, with dielectric constants of ∼11–13. The novelty of this work lies in the green development of rare Mo-based materials that uniquely combine structural, electrical, and catalytic features. Importantly, this study establishes correlations between these properties, representing, to the best of our knowledge, one of the first systematic investigations of molybdenum coordination complexes incorporating thiophene–carbohydrazide ligands.

## Abstract 2

This study focuses on the synthesis of Mo complexes coordinated with a thiophene-2-carboxylic acid hydrazone-type ligand (H_2_L), obtained *via* mechanochemical reaction condensation of 2-hydroxybenzaldehyde with thiophene-2-carboxylic acid hydrazide.

## Graphical abstract

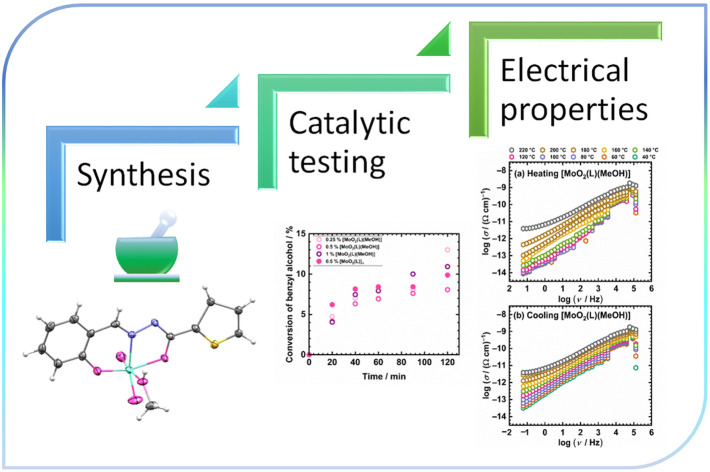

## 1 Introduction

Hydrazides, carbohydrazides, and structurally related analogs are well-established, versatile synthetic intermediates for constructing diverse heterocyclic systems. A substantial body of literature has demonstrated that numerous heterocyclic carbohydrazides and their derivatives exhibit pronounced and wide-ranging biological activities.^1,2^ Although several transition metal complexes involving 2-thiophenecarboxylic hydrazide as a bidentate ligand have been reported, particularly with vanadium, zinc, manganese, cobalt, and nickel,^3,4^ the corresponding hydrazone derivatives remain comparatively underexplored. Only a limited number of complexes, mainly with cobalt, nickel, copper, and zinc, have been synthesised and investigated, with most studies emphasising their biological activity and pharmacological potential.^5,6^ In contrast, their physicochemical properties and broader applications remain less systematically examined. Notably, a highly sensitive and selective fluorescent chemosensor for Al^3+^ ions, based on a Schiff base derived from thiophene-2-carboxylic acid hydrazide, has been reported, underscoring the potential of such ligands in analytical and coordination chemistry.^7^ This highlights a promising, but relatively untapped, pathway for expanding their role in sensing technologies, catalysis, and materials science. On the other side, mechanochemistry, which uses mechanical energy to drive chemical transformations, offers a green and sustainable alternative to traditional solvent-based synthesis by minimizing or eliminating solvent use.^8,9^ Grinding methods, under solvent-free or liquid-assisted conditions (LAG), enable efficient ligand and metal complex preparation, promoting the green chemistry principles.^10,11^

**Scheme 1:**
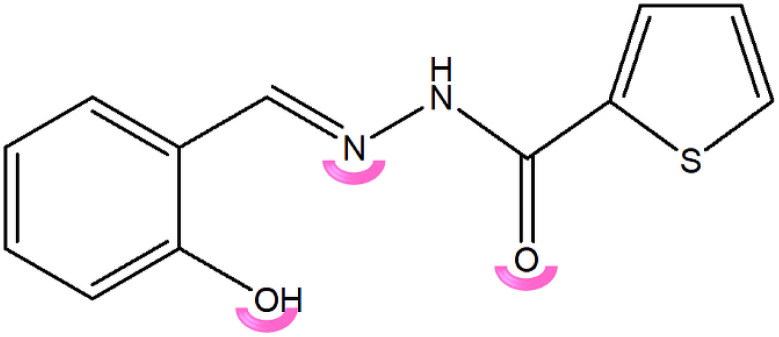
Ligand used for the preparation of Mo-complexes. The pink arc block represents the coordination atoms.

Based on these considerations, we directed our investigation toward the mechanochemical synthesis of molybdenum complexes derived from 2-thiophenecarboxylic hydrazone, with the dual objective of evaluating their catalytic and electrical properties. To assess the catalytic potential, benzyl alcohol oxidation was selected as a model transformation owing to its significance as a benchmark reaction in oxidation catalysis research. Despite the development of numerous oxidants and oxidation methodologies for such transformations, achieving the oxidation of organic substrates using environmentally benign oxidants, under mild reaction conditions, remains a compelling and unresolved challenge in this field^12,13^ In parallel, our preliminary studies on the electrical behavior of Mo-based materials have revealed promising semiconducting characteristics, motivating a more systematic exploration of their potential applications in the field of electronic materials.^14,15^ Therefore, the present work aims to bridge these two aspects by developing novel Mo-hydrazone complexes, with thiophene-2-carbohydrazide type ligand (Scheme 1) and elucidating their structure–property relationships concerning both catalytic efficiency and electronic performance.

## 2 Results and discussion

### 2.1 Preparation, spectroscopic characterization and thermal stability study

The starting ligand was prepared by a LAG mechanochemical reaction between salicylaldehyde and thiophene-2-carbohydrazide, with the addition of methanol, as well as the classic synthesis in MeOH. The purity of the prepared ligand was examined using differential scanning calorimetry (DSC). The DSC curve (SI, Fig. S1) shows a single endothermic minimum at 187 °C, followed by exothermic decomposition. The ligand melted in the temperature range of 183–192 °C. The enthalpy of fusion was determined to be +352.8 mJ, and based on the known amount of ligand, the molar enthalpy of fusion was calculated to be +11.8 kJ mol^−1^. The DSC curve shows another, less intense minimum at a temperature slightly above 187.0 °C. This may be attributed either to traces of unreacted thiophene-2-carbohydrazide in the ligand sample or to the presence of polymorphic forms of the ligand. The literature value for the melting point range of thiophene-2-carbohydrazide is 136–139 °C, which suggests that the ligand is not contaminated by hydrazide, and potentially implies the existence of a polymorph.

The IR spectrum of the ligand H_2_L (SI, Fig. S2) shows a characteristic band at 1656 cm^−1^, indicating the presence of a keto group, while the band at 1606 cm^−1^ confirms the formation of an imine bond (–C=N_imine_). Furthermore, aromatic stretching vibrations of C–H, C=C, and C–O_phenol_ are present, appearing at 3067, 1490, and 1250 cm^−1^, respectively. Additionally, N–H stretching is observed at 3257 cm^−1^. Table 1 presents a comparison of the positions of characteristic bands in the IR spectrum of the ligand with those in the spectra of 2-hydroxybenzaldehyde and thiophene-2-carbohydrazide.

**Table 1:** Comparison of the positions of characteristic bands in the IR spectrum of the ligand with those in the spectra of 2-hydroxybenzaldehyde and thiophene-2-carbohydrazide

Characteristic group	2-Hydroxy benzaldehyde	Thiophene-2-carbohydrazide	H_2_L ligand
O–H	3400–2600	—	3400–2600
–N–H	—	3310	3257
=C_ar_–H	3059	3073	3067
–C=O_keto_	1660	1616	1656
–C=N_imin_	—	—	1606
–C=C_ar_	1485	1537	1490
–C–O_fenol_	1270	—	1250

To ensure accurate species identification, all complexes were initially synthesized using a conventional solution-based method (the reaction of [MoO_2_(acac)_2_] and H_2_L in the appropriate solvent) and subsequently reproduced using LAG mechanochemistry. This dual approach not only confirmed the reproducibility and structural consistency of the prepared complexes but also highlighted the efficiency of the mechanochemical route as a sustainable alternative to classical synthesis. The compounds obtained were compared by PXRD method, to confirm the same species obtained. The complexes were characterised by IR-ATR spectroscopy, Table 2 and SI, Fig. S3–S5. In all complexes, the bands corresponding to –N–H and –C=O_keto_ stretching, which appear in the IR spectrum of the H_2_L ligand at 3257 cm^−1^ and 1656 cm^−1^, respectively, are absent. This observation indicates that in all complexes, the hydrazone ligand is coordinated to the Mo(vi) centre in its enol-imine form, which arises from keto–enol tautomerism in solution (–C=N–NH–(C=O)– ⇄ C=N–N=(C–OH)–), followed by deprotonation of the ligand.

**Table 2:** Comparison of the positions of characteristic bands in the IR spectrum of the obtained Mo complexes

Characteristic group	[MoO_2_(L)(MeOH)]	[MoO_2_(L)(H_2_O)] *ν̃*/cm^−1^	[MoO_2_(L)]_*n*_
O–H	3400–2600	3600–2800	—
–N–H	—	—	—
=C_ar_–H	3097	3093	3029
H_2_O	—	1653	—
–C=N_imin_	1597	1598	1600
–C=C_ar_	1493	1493	1445
–C–O_phenol_	1271	1268	1268
MeOH	1010	—	—
*cis*-{MoO_2_^2+^}	930, 910	940, 910	925
O=Mo⋯O	—	—	850
C–S	639	638	640

The characteristic band in the spectra of the complex obtained from MeOH, a yellow-coloured one, showed an absorption maximum at 1010 cm^−1^, corresponding to methanol, implying the coordination of MeOH at the sixth coordination site of the Mo(vi) centre, assuming the formula of the obtained complex [MoO_2_(L)(MeOH)]. Furthermore, two strong absorption maxima at 930 and 910 cm^−1^ correspond to the symmetric and asymmetric stretching modes characteristic of the *cis*-{MoO_2_^2+^} units. The IR spectrum of the complex obtained from acetonitrile, an orange-coloured one, showed a broad band in the range of 3600–2800 cm^−1^, corresponding to O–H stretching and implying the general formula of the complex to be [MoO_2_(L)(H_2_O)]. The bands at 940 cm^−1^ and 910 cm^−1^ correspond to the *cis*-MoO_2_^2+^ core. However, after standing for several weeks, the colour of the complex gradually changed from yellow to reddish-brown, suggesting a possible transformation of the complex. The IR spectrum of the resulting brown product closely matched that of the complex obtained from dichloromethane, indicating that the same or a structurally similar species was formed. In the obtained complex, the characteristic band at 850 cm^−1^ implies that the linkage of monomeric units occurs through O=Mo⋯O=Mo interactions.^16,17^ This finding is consistent with literature data, which report that the characteristic vibrational bands associated with polymerisation *via* terminal oxygen atoms in polynuclear compounds typically appear in the 850–800 cm^−1^ region. Additionally, the absorption band at 925 cm^−1^ corresponds to Mo=O stretching vibrations. The general formula of the obtained complex is [MoO_2_(L)]_*n*_.

**Fig. 1:**
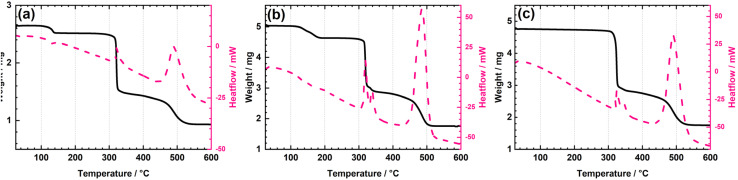
TG/DSC curves for the complexes: (a) [MoO_2_(L)(MeOH)], (b) [MoO_2_(L)(H_2_O)] and (c) [MoO_2_(L)]_*n*_. The black curve represents mass loss, while the pink one is heat flow.

Based on the thermograms of the complexes synthesised in solution, Fig. 1, it can be concluded that, in cases where solvent molecules are coordinated, the initial mass loss corresponds to solvent release. For instance, the methanol release for the complex [MoO_2_(L)(MeOH)] occurs in the range 119–178 °C, while the water release for the complex [MoO_2_(L)(H_2_O)] is narrower, in the interval 119–133 °C. This is followed by oxidative decomposition, [MoO_2_(L)(MeOH)] (327–519 °C), [MoO_2_(L)(H_2_O)] (314–508 °C), yielding MoO_3_, further confirmed by the comparison of the residue and the commercially available MoO_3_. Each thermogram also includes a differential scanning calorimetry (DSC) curve, which provides complementary information and allows for the differentiation between endothermic and exothermic processes. Solvent loss steps are endothermic, whereas oxidative decomposition of the intermediate formed after solvent removal occurs through multiple exothermic events. The thermal decomposition of the complex [MoO_2_(L)]_*n*_ occurs in one step, at 329–530 °C.

Table 3 presents a comparison of the experimental and theoretical mass fractions of methanol, water, and MoO_3_ in the complexes synthesised in solution. The agreement between experimental and theoretical values supports the proposed molecular formulas of the obtained complexes.

**Table 3:** Results of TGA analysis

Complex	(*w*_exp_/%)/(*w*_t_/%)		
	MeOH	H_2_O	MoO_3_
[MoO_2_(L)(MeOH)]	8.84 (7.92)	—	33.57 (35.61)
[MoO_2_(L)(H_2_O)]	—	4.79 (4.62)	35.69 (36.89)
[MoO_2_(L)]_*n*_	—		36.82 (38.67)

*In situ* synthesis (reaction of [MoO_2_(acac)_2_], thiophene-2-carbohydrazide and 2-hydroxybenzaldehyde, 1 : 1 : 1) in methanol resulted in [MoO_2_(L)(MeOH)] complex, while the reaction in acetonitrile provided [MoO_2_(L)]_*n*_ and not water-coordinated complex. The same results were obtained by mechanochemical synthesis as well. Although both solution-based and *in situ* syntheses are performed in acetonitrile, subtle differences in reaction dynamics, water content, and ligand formation timing likely govern the outcome. In solution-based synthesis, even minimal water can stabilise the mononuclear [MoO_2_(L)(H_2_O)] complex. In contrast, *in situ* and mechanochemical synthesis, favours the formation of polynuclear [MoO_2_(L)]_*n*_ complex through Mo=O⋯Mo bridging. An additional experiment was performed with the complex [MoO_2_(L)(MeOH)]. It was heated to 200 °C, and its color changed to brown-red. The IR spectra showed that the mononuclear complex transformed to the polynuclear one.

### 2.2 Insights into crystal and molecular structure of ligand H_2_L and Mo(vi) complexes

Single-crystal X-ray diffraction (SCXRD) analyses were performed on three compounds (SI, Tables S1–S3): the free ligand H_2_L and two Mo(vi) complexes, [MoO_2_(L)(MeOH)] and [MoO_2_(L)(H_2_O)]·(CH_3_)_2_CO. Suitable single crystals of H_2_L and [MoO_2_(L)(MeOH)] were obtained directly from solution, whereas crystals of [MoO_2_(L)(H_2_O)]·(CH_3_)_2_CO were isolated by recrystallisation from acetone.

The hydrazone ligand H_2_L (SI Fig. S6) crystallises in the expected keto-amino tautomeric form, as confirmed by characteristic bond distances (SI Table S2). The molecular structure is stabilised by an intramolecular O–H⋯N=C hydrogen bond between the aryl hydroxyl and the imine nitrogen. In the crystal packing, additional intermolecular N–H⋯O–C hydrogen bonds link adjacent ligand molecules into one-dimensional zig-zag chains (SI Fig. S6(c)).

The studied complexes, [MoO_2_(L)(MeOH)] and [MoO_2_(L)(H_2_O)]·(CH_3_)_2_CO, exhibit geometrical features that are common throughout the class of reported dioxomolybdenum(vi) species (SI Fig. S7 and S8).^14,15,38^ In both cases, H_2_L is coordinated in its doubly deprotonated form, with deprotonation occurring at the aryl hydroxyl and hydrazone amide sites, consistent with the enol-imine tautomer. The metal center adopts a distorted octahedral geometry, in which the ONO donor set of the ligand occupies three equatorial positions, while the *cis*-dioxo unit completes the equatorial plane. The axial site is occupied by a solvent molecule (methanol or water). Although the primary coordination environments are analogous, the supramolecular association and crystal packing differ markedly, depending on the identity of the coordinated solvent molecule.

**Fig. 2:**
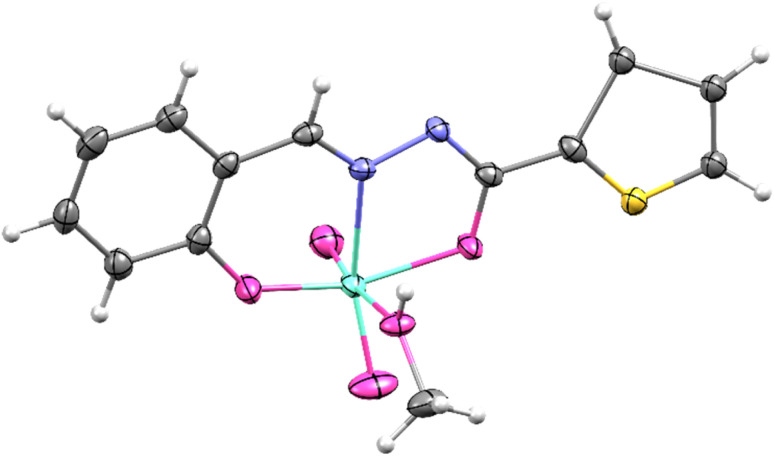
Molecular and crystal structure of [MoO_2_(L^1^)(MeOH)]. The displacement ellipsoids are drawn at a 50% probability level at 100 K. Hydrogen atoms are presented as spheres of arbitrary, small radii.

In the methanol-coordinated complex [MoO_2_(L)(MeOH)], a single hydrogen-bond donor is available. The molecules are linked through intermolecular O–H_(methanol)_⋯N_(imine)_ interactions, giving rise to 1D zig-zag chains propagating through the crystal lattice (SI Fig. S7(b)). In contrast, the water-coordinated complex [MoO_2_(L)(H_2_O)]·(CH_3_)_2_CO possesses two hydrogen-bond donors. Here, supramolecular homodimers are generated *via* O–H_(water)_⋯N_(imine)_ interactions, while the second donor participates in O–H_(water)_⋯O=C_(acetone)_ hydrogen bonding. These interactions connect the dimers into 1D linear chains (SI Fig. S8(c)). The observed hydrogen-bonding motifs for both methanol- and water-coordinated species are consistent with those reported for related dioxomolybdenum(vi) complexes (Fig. 2).^15^

### 2.3 Catalytic performance

The oxidation of benzyl alcohol was carried out using a 30% solution of hydrogen peroxide as the oxidant, with the addition of acetonitrile as a co-solvent. The addition of a small amount of acetonitrile enhances the oxidation of the substrate, potentially attributed to the formation of a reactive intermediate, through the reaction between acetonitrile and hydrogen peroxide.^18^ This reactive species was characterised in the context of catalytic oxidation reactions of tertiary amines using aqueous hydrogen peroxide, where it was observed that the addition of a small quantity of acetonitrile improved key reaction parameters, such as conversion and selectivity. Two Mo(vi) complexes were investigated as potential catalysts: [MoO_2_(L)]_*n*_ and [MoO_2_(L)(MeOH)]. [MoO_2_(L)(H_2_O)] was excluded from catalytic evaluation because of its low yield and its inconsistency with green chemistry principles, as it was synthesized through a solution-based pathway. The catalytic reactions were conducted using low catalyst loading, and a total of four reactions were carried out. Three of them involved the mononuclear complex [MoO_2_(L)(MeOH)], with varying catalyst loadings of 0.25 mol%, 0.5 mol%, and 1 mol%. The fourth reaction employed the polynuclear complex [MoO_2_(L)]_*n*_ at a catalyst loading of 0.5 mol%. In all reactions conducted, the molybdenum complex was fully dissolved, indicating that all catalytic oxidations proceeded under homogeneous conditions. Upon addition of either the mononuclear complex [MoO_2_(L)(MeOH)] or the polynuclear species [MoO_2_(L)]_*n*_, the reaction mixture exhibited a gradual colour change, from red orange, through yellow orange, to a progressively darker orange solution.

The reaction temperature was set to 70 °C. This temperature selection is supported by literature data on the catalytic oxidation of linear alcohols with hydrogen peroxide as an oxidant. Titanium silicate was employed as catalyst and at reaction temperature of 70 °C, the conversion and TOF were the highest.^19^ Furthermore, Cr(iii) imine complexes as homogeneous catalysts for benzyl alcohol oxidation have showed increase of alcohol conversion at 70 °C, and the aldehyde selectivity was around 100%.^20^ Moreover, the addition of MeCN and the temperature of 70 °C positively influenced benzyl alcohol conversion and aldehyde selectivity with heteropoly tungstate catalysts.^21^

The catalytic activity of the two investigated Mo(vi) complexes was evaluated in terms of the conversion of benzyl alcohol and selectivity toward benzaldehyde. Based on the data in Table 4, it can be generally stated that the conversion values of benzyl alcohol were similar across all four catalytic reactions, ranging from 8 to 13%, indicating relatively consistent catalytic performance under the tested conditions.

**Table 4:** Catalytic parameters calculated for the tested catalysts

Catalyst	Con^d^/%	Sel^e^/%	TON^f^	TOF^g^
[MoO_2_(L)(MeOH)]^a^	8.07	82.44	15.98	24.71
[MoO_2_(L)]_*n*_^a^	9.87	74.78	18.62	35.14
[MoO_2_(L)(MeOH)]^b^	13.01	51.37	50.70	55.33
[MoO_2_(L)(MeOH)]^c^	10.93	72.08	10.28	11.40

a Reaction conditions: time, 120 min; temperature, 70 °C; molar ratio [Mo]/benzyl alcohol/oxidant: 0.1/20/40.

b Reaction conditions: time, 120 min; temperature, 70 °C; molar ratio [Mo]/benzyl alcohol/oxidant: 0.05/20/40.

c Reaction conditions: time, 120 min; temperature, 70 °C; molar ratio [Mo]/benzyl alcohol/oxidant: 0.2/20/40.

d Benzyl alcohol converted at the end of the reaction.

e Molar ratio of benzaldehyde to converted benzyl alcohol at the end of the reaction.

f Moles of converted benzyl alcohol per mole of catalyst at the end of the reaction.

g Moles of converted benzyl alcohol per mole of catalyst per hour, measured after 20 minutes of reaction (TOF).

**Fig. 3:**
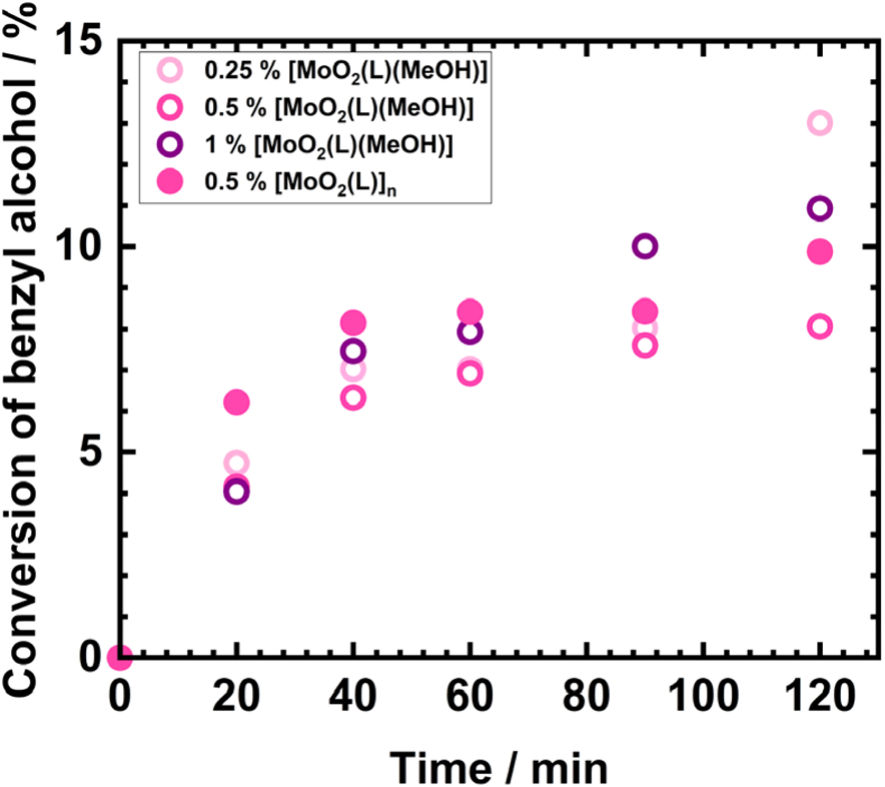
Kinetic profiles for benzyl alcohol oxidation with Mo-catalysts.

Compared to conversion values, catalyst selectivity toward benzaldehyde exhibits greater variability and follows a different trend depending on both catalyst loading and structure. An especially noteworthy result is observed for the mononuclear methanol complex [Mo] at a loading of 0.5 mol%, which achieved the highest aldehyde selectivity at the end of the reaction (82.44%), while mononuclear catalyst with 0.25 mol% exhibited the lowest selectivity toward the product, benzaldehyde, 51.37%. It seems that low catalyst loading is more prone to further overoxidation processes. Conversely, increasing the catalyst loading of [MoO_2_(L)(MeOH)] to 1 mol% led to selectivity of 72.08%. The polynuclear complex [MoO_2_(L)]_*n*_ at a 0.5 mol% loading exhibits a selectivity toward benzaldehyde of 74.78%, which is comparable to the mononuclear complex [MoO_2_(L)(MeOH)] at 1 mol% loading. During catalytic reactions using the mononuclear complex [MoO_2_(L)(MeOH)], both catalyst structure and loading were found to influence catalytic performance. As shown in the kinetic profile in Fig. 3, benzyl alcohol conversions were nearly identical for the 0.25 mol% and 0.5 mol% loadings up to 90 minutes, implying similar early-time active species. In contrast, the 1 mol% catalyst loading consistently led to higher conversion throughout the reaction. Interestingly, at the end of the reaction (120 minutes), the highest conversion (13.01%) was achieved using the lowest catalyst loading (0.25 mol%) of [MoO_2_(L)(MeOH)]. This observation suggests that catalyst loading affects Mo(vi) catalyst activity in terms of conversion. However, this effect is modest within the 0.25–1 mol% range, as the conversion values for all three loadings differ by less than 5% at all sampling times. When examining the effect of catalyst structure on conversion, the polynuclear complex [MoO_2_(L)]_*n*_ initially exhibits higher activity than the mononuclear methanol complex, up to 60 minutes. After this point, the influence of catalyst loading becomes more pronounced: [MoO_2_(L)]_*n*_ at 0.5 mol% loading becomes less active than the [MoO_2_(L)(MeOH)] complex at 0.25 and 1 mol%, but more active than the methanol complex at 0.5 mol%. Specifically, the polynuclear complex at 0.5 mol% achieves a benzyl alcohol conversion of 10%, compared to 8 and 11% for the methanol complex at 0.5 and 1 mol% loadings, respectively.

**Fig. 4:**
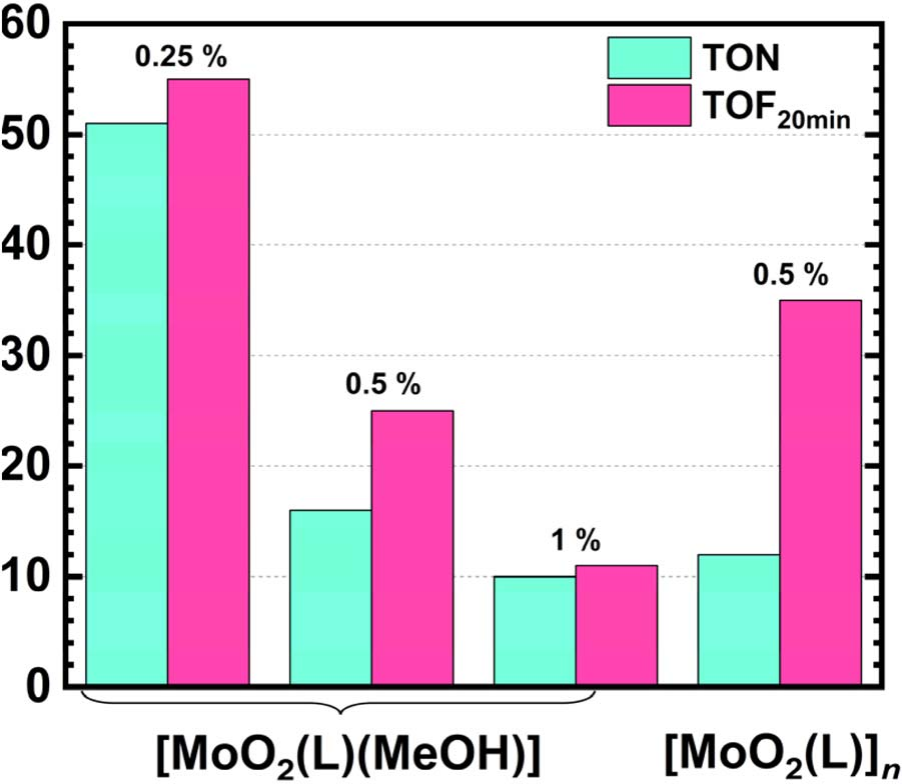
TOF *vs.* TON parameter for the tested Mo-catalysts.

Based on the TOF (Turnover Frequency) values presented in Table 4 and Fig. 4, the highest initial catalytic activity was observed for the mononuclear methanol complex [MoO_2_(L)(MeOH)] at a [Mo] loading of 0.25 mol%, with a TOF of 55.33 h^−1^. This is followed by the polynuclear complex [MoO_2_(L)]_*n*_, which demonstrated greater activity compared to the mononuclear methanol complex at both the same ([Mo] 0.5 mol%) and higher ([Mo] 1 mol%) loadings. The initial TOF for the polynuclear complex was 35.14 h^−1^, whereas the mononuclear complex at 0.5 mol% and 1 mol% loadings showed TOF values of 24.71 h^−1^ and 11.40 h^−1^, respectively. The increase in catalytic activity with decreasing catalyst loading can be attributed to the faster dissolution and more rapid formation of the catalytically active species. The influence of the catalyst structure on benzyl alcohol conversion and initial catalytic activity is also explained by the faster generation of the active pentacoordinated species [MoO_2_(L)]. In the case of the polynuclear complex [MoO_2_(L)]_*n*_, the dissociation of the repeating units appears to require less energy than the cleavage of the methanol ligand coordinated at the sixth position of the Mo(vi) center in the mononuclear complex [MoO_2_(L)(MeOH)]. Consequently, the polynuclear complex can readily form the active species and contribute to a higher catalytic activity in the early stages of the reaction.

Comparable catalytic performance parameters for the oxidation of benzyl alcohol to benzaldehyde have been reported for first-row transition metal complexes bearing various hydrazone ligands.^22^ In the reported study, the catalytic activities of Cu(ii), Ni(ii), and Co(ii) complexes with a Schiff base ligand, 2,2′-(propenylenedioxy)dibenzaldehyde bis (thiosemicarbazone), were evaluated using different oxidants: *tert*-butyl hydroperoxide (TBHP), hydrogen peroxide (H_2_O_2_), and *meta*-chloroperoxybenzoic acid (*m*-CPBA).

The Cu(ii) and Co(ii) complexes were studied using TBHP in water as the oxidant, yielding conversion and selectivity values of approximately 60% for Cu(ii), and 65% conversion with 59% selectivity for Co(ii). The Ni(ii) complex demonstrated generally higher catalytic activity. Initially, the effect of different oxidants was examined at a fixed catalyst loading of 0.1%, resulting in the following conversion and selectivity values: with *m*-CPBA in DMF: 45% conversion, 35% selectivity; with H_2_O_2_ in water: 26% conversion, 57% selectivity, and with TBHP in water: 89% conversion, 70% selectivity. As TBHP in water provided the most favorable results, further studies on the catalytic activity with varying Ni(ii) complex loadings were conducted using this oxidant. At a catalyst loading of 0.067%, a conversion of 93% and a selectivity of 65% were achieved. At the lowest tested loading of 0.05%, the conversion increased slightly to 95%, while selectivity decreased to 60%. When comparing these findings to the catalytic behavior of the Mo(vi) complexes investigated in this study, the closest match is observed with the Ni(ii) complex at 0.1% loading and H_2_O_2_ in water as the oxidant. The conversion of 26% obtained under those conditions is approximately 13% higher than the maximum conversion observed in this study for the [MoO_2_(L)(MeOH)] catalyst at 0.25% loading, while the selectivity of 57% for the Ni(ii) complex is comparable to the 51.37% observed for the Mo(vi) complex.

Furthermore, a similar trend of increasing benzyl alcohol conversion accompanied by a decrease in selectivity toward benzaldehyde with decreasing catalyst loading is evident in both, the Ni(ii) and [MoO_2_(L)(MeOH)] systems. Further comparison was done with [MoO_2_(L)(H_2_O)] catalyst, where L is deprotonated form of the ligands obtained by the reaction of 3-ethoxysalicylaldehyde with 4-aminobenzohydrazide.^23^ The reported catalyst (0.6 mol%) was tested in benzyl alcohol oxidation, with H_2_O_2_ and MeCN, and aldehyde yiled ofter 2 h of the reaction was 92%. Another example is [MoO_2_(L)(EtOH)], where L is deprotonated form of the ligand obtained from nicotinic hydrazide with 5-nitrosalicylaldehyde.^24^ With oxidant urea hydrogen peroxide and 10 mL of MeCN aldehyde yield was 76% after 2 h of the reaction. Two similar Mo catalysts from our research group were also tested for benzyl alcohol oxidation, [MoO_2_(VIH)]_4_ and [MoO_2_(VIH)(EtOH)], where VIH is deprotonated form of the ligand obtained by the reaction of 3-methoxy-2-hydroxybenzaldehyde and isonicotinyl hydrazine.^25^ The reaction was performed using different oxidants: TBHP in water and decane, and H_2_O_2_ with MeCN. After 5 h of the reaction, benzyl alcohol conversion was 11% for [MoO_2_(VIH)]_4_ and very low for [MoO_2_(VIH)(EtOH)], while aldehyde selectivity for [MoO_2_(VIH)]_4_ was 90%. Further, [MoO_2_(SIH)]_*n*_, where SIH is deprotonated form of the ligand prepared by the reaction of salicylaldehyde and isonicotinic acid hydrazide, catalysed the benzyl alcohol oxidation with H_2_O_2_ and MeCN.^26^ After 2 h of the reaction conversion was 11% and the aldehyde selectivity 90%. In all the reported studies, the molybdenum catalysts are soluble in the reaction medium and therefore cannot be reused. The catalytic parameters observed in those studies, including TOF and TON values, are comparable to those obtained in the present study (SI, Table S4).

A plausible mechanism has been suggested (SI, Fig. S9, S10 and Table S5).

### 2.4 Solid-state impedance spectroscopy studies

The electrical/dielectric properties of molybdenum complexes were explored using solid-state impedance spectroscopy (ss-IS), technique applied to study electrical transport in different materials, amorphous and/or crystalline ones.^27–30^

The results of the electrical and dielectric properties of Mo(vi) complexes obtained *via in situ* synthesis in acetonitrile [MoO_2_(L)]_*n*_, and in methanol [MoO_2_(L)(MeOH)] are presented below. Based on the thermal stability data, a heating–cooling cycle was conducted (10 °C per step). The samples were heated from 30 °C to 220 °C and subsequently cooled back down to 30 °C. The upper temperature limit (220 °C) is in accordance with the TGA results, which indicates that the coordinated molecule is released from the complex structures above this temperature. This release is believed to induce a structural transformation into a polynuclear complex that remains stable up to ∼250 °C.

**Fig. 5:**
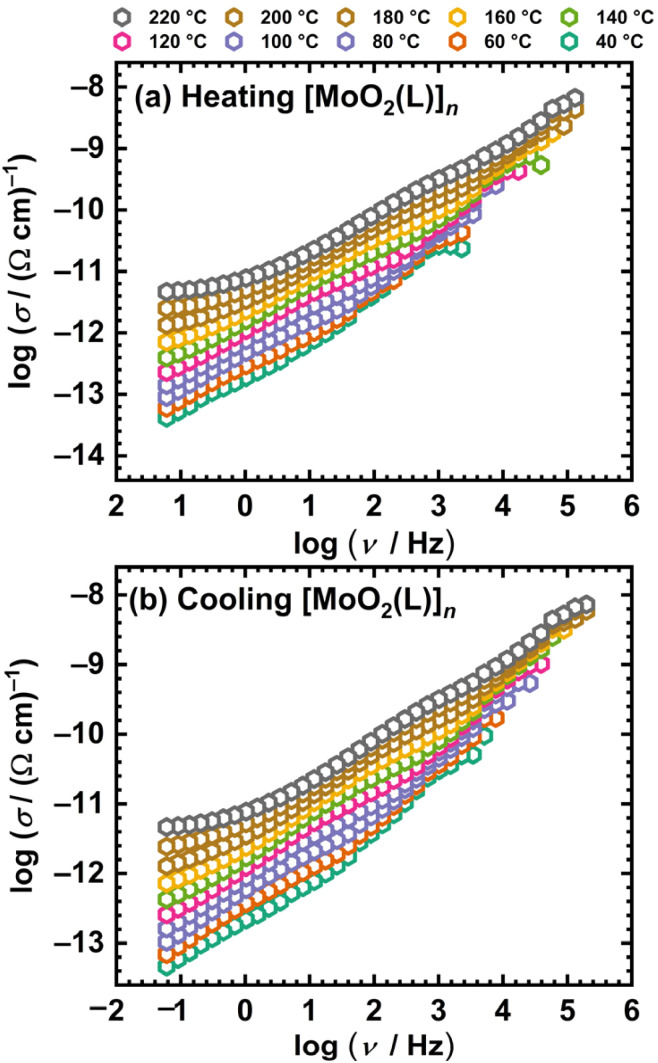
Conductivity isotherms at different temperatures for [MoO_2_(L)]_*n*_ in heating (a) and cooling (b) run.

Fig. 5 shows the frequency dependence of the real part of the electrical conductivity, *σ*′, for the polynuclear complex [MoO_2_(L)]_*n*_ at various temperatures, *i.e.*, the conductivity spectra. For clarity, the spectra of the complexes are presented in the temperature range of 40–220 °C in 20 °C increments.

For both investigated samples, conductivity is a thermally activated process and exhibits semiconducting behavior, increasing with temperature. Furthermore, Fig. 5 shows that at high temperatures and low frequencies, the conductivity becomes frequency-independent, exhibiting a so-called DC plateau corresponding to direct current (DC) conductivity. Conversely, in the high-frequency region, conductivity shows frequency dependence, which corresponds to the alternating current (AC) conductivity (dispersive region). The transition from the DC to the AC region shifts toward higher frequencies as the temperature increases. Owing to the low conductivity of the Mo(vi) complexes under study, the DC conductivity value could not be directly read from the graph for most temperatures. Therefore, experimental complex impedance spectra were analysed by modelling, see Experimental part.

For the sample [MoO_2_(L)]_*n*_, the impedance spectrum, so-called Nyquist plot, is shown in the SI, Fig. S11. The experimental data are presented in the complex plane as the dependence of the imaginary component (*Z*″) *vs.* the real component (*Z*′). The spectrum forms a semicircular shape and was analysed using a simple equivalent electrical circuit (EEC) model based on a parallel *R*–CPE configuration, where *R* represents a resistor corresponding to the sample's resistance, and the constant phase element (CPE) approximates the sample's capacitance. A good overlap was observed, indicating a strong agreement between experimental data and theoretical model. The sample's resistance at a given temperature can be determined from the intersection of the fitted semicircle with the *x*-axis.

For the [MoO_2_(L)]_*n*_ sample, the resistance value @200 °C is 3.22 × 10^11^ Ω, while from that, the DC conductivity was calculated to be 2.09 × 10^−12^ (Ω cm)^−1^. Since the [MoO_2_(L)]_*n*_ sample is a polynuclear complex that does not contain solvent molecules, ss-IS revealed no changes during the heating–cooling cycle, which is consistent with TG analysis, Fig. 1(c), and single-step decomposition observed. The sample remains in its polynuclear form throughout entire temperature range of ss-IS setup.

**Fig. 6:**
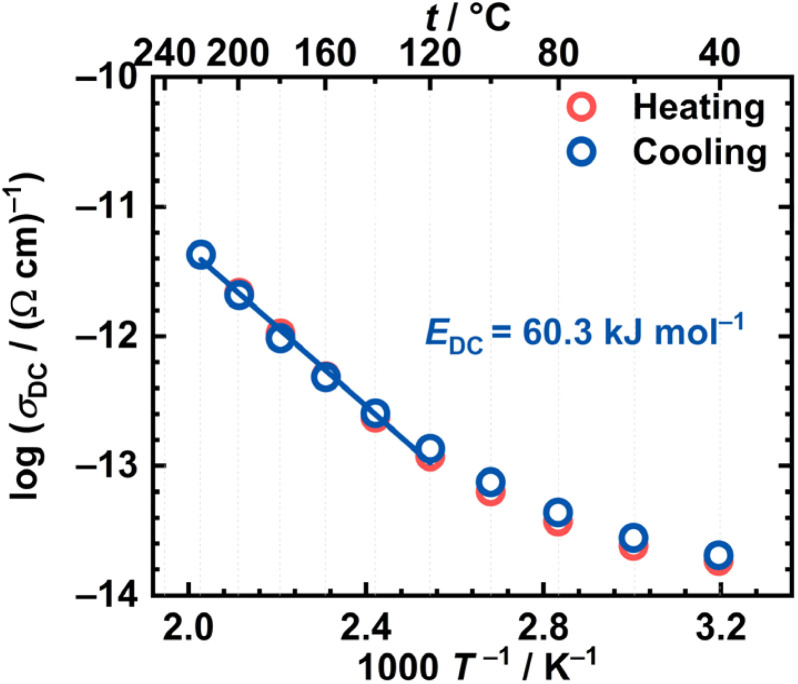
Arrhenius plot—temperature dependence of DC conductivity (log(*σ*_DC_) *vs.* 1000/*T*) for both runs (red circle—heating, blue circle—cooling) for [MoO_2_(L)]_*n*_.

As mentioned, the DC conductivity of the [MoO_2_(L)]_*n*_ sample shows a temperature dependence, indicating a thermally activated process following the Arrhenius behaviour. The activation energy for the DC conduction process, *E*_DC_, can be determined from the slope of linear dependence log(*σ*_DC_) *vs.* 1000*T*^−1^/K^−1^, using the following expression:1
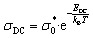
where *σ*_DC_ is the DC conductivity, 
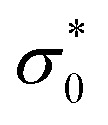
 is the pre-exponential factor, *k*_B_ is the Boltzmann constant, and *T* is the temperature in K. The Arrhenius plot for the [MoO_2_(L)]_*n*_ sample is shown in Fig. 6, while calculated activation energies with other electrical parameters are summarised in Table 5 for both samples. Values for *E*_DC_ are in the range ∼60–63 kJ mol^−1^ which is in line with values reported in the literature for various complexes (metal, ligand) along with those seen in a variety of semiconductive materials^14,15,31–39^ with dominant electronic transport mechanisms as in our case. Comparison of the heating and cooling cycles of the [MoO_2_(L)]_*n*_ sample reveals only slight differences, which further confirms that no structural transformation occurs within investigated temperature range.

**Table 5:** Electrical parameters for the obtained Mo complexes at 180 °C in the cooling cycle

Sample	*σ*_DC_/(Ω cm)^−1^ (cooling cycle)	*E*_DC_/kJ mol^−1^ (cooling cycle)
[MoO_2_(L)]_*n*_	9.66 × 10^−13^	60.3
[MoO_2_(L)(MeOH)]	9.17 × 10^−13^	62.8

From the conductivity spectra (Fig. 5) and the Arrhenius plot (Fig. 6), it is evident that the temperature dependence is weak below 100 °C, while above this temperature, significant temperature dependence emerges. The activation energy was determined for the high-temperature range during the cooling cycle, where conductivity exhibits a clear linear temperature dependence. The obtained activation energy value (60.3 kJ mol^−1^) suggests that the polynuclear complex, in which the monomeric units are connected *via* O=Mo⋯O=Mo interactions, enables significant and uninterrupted charge-carrier transport along the entire polynuclear structure. It is in good agreement with literature-reported activation energies for Mo(vi) polynuclear complexes with various hydrazone ligands, such as the ligand derived from 2-hydroxy-5-nitrobenzaldehyde and 2-hydroxybenzohydrazide^38,39^

**Fig. 7:**
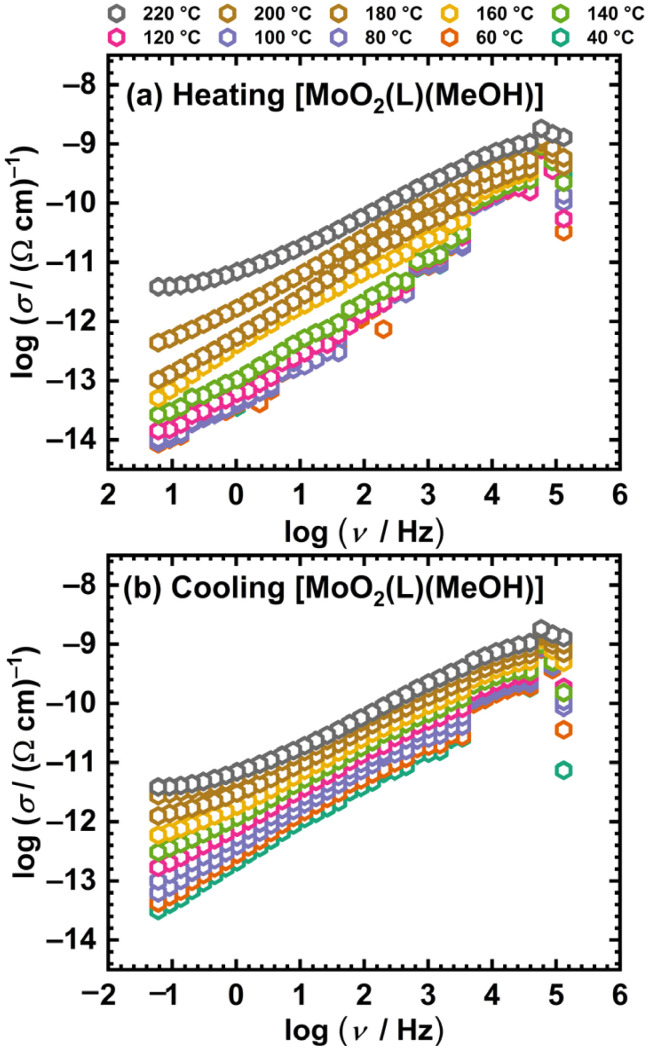
Conductivity isotherms at different temperatures for [MoO_2_(L)(MeOH)] in heating (a) and cooling (b) runs.

Opposed to [MoO_2_(L)]_*n*_, the mononuclear methanol complex shows distinct differences in the conductivity spectra between the heating and cooling cycles, see Fig. 7.

The conductivity spectra of the [MoO_2_(L)(MeOH)] complex exhibits a non-monotonic temperature dependence in the heating cycle, resulting from the increase in conductivity due to the release of coordinated methanol and the presumed transformation into a polynuclear species. The thermogram of the complex, see Fig. 7(a), indicates that methanol is released in the temperature range of ∼80 °C to ∼190 °C, while during the heating cycle of ss-IS, weak temperature dependence is observed up to ∼100 °C, corresponding to partial methanol release.

Above 100 °C, significant methanol loss occurs, accompanied by the expected transformation of the initial sample into the polynuclear [MoO_2_(L)]_*n*_ species, reflected in the non-monotonic change in conductivity. In contrast, the cooling cycle displays isotherms which show no indication of abrupt changes in conductivity, consistent with the expectation that the transformed species is stable, see Fig. 7(b). Below 100 °C, a weak temperature dependence of conductivity is observed.

**Fig. 8:**
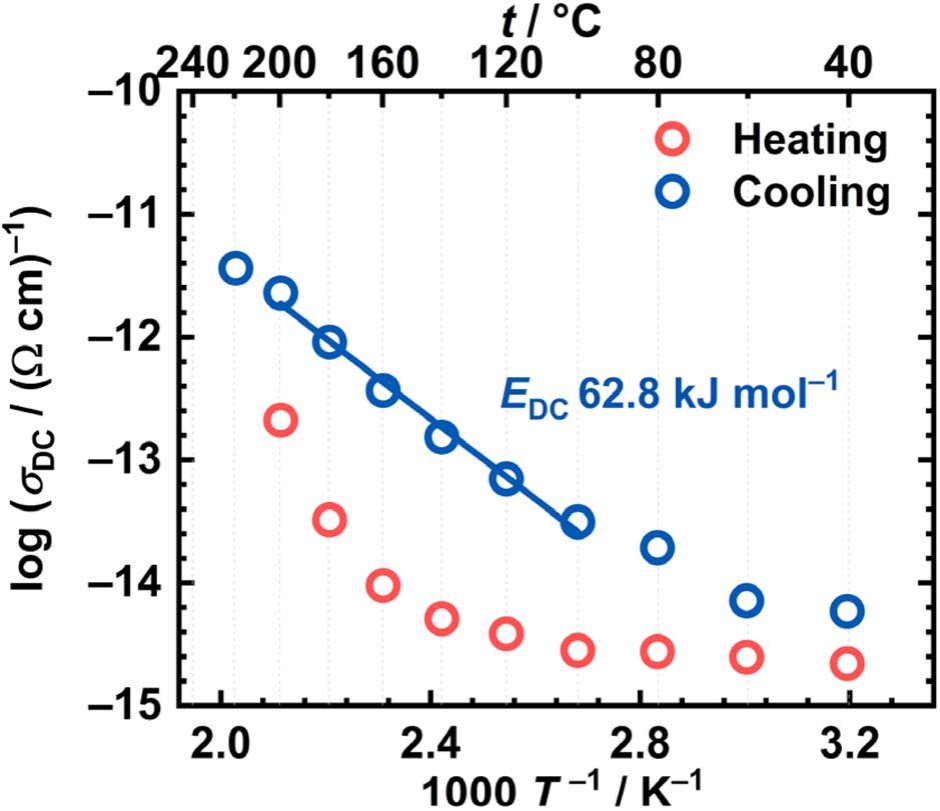
Arrhenius plot—temperature dependence of DC conductivity (log(*σ*_DC_) *vs.* 1000/*T*) for both runs (red circle—heating, blue circle—cooling) for [MoO_2_(L)(MeOH)].

The difference between the heating and cooling cycles of the [MoO_2_(L)(MeOH)] complex is also evident from the Arrhenius plot, see Fig. 8. In comparison to [MoO_2_(L)]_*n*_, the [MoO_2_(L)(MeOH)] sample exhibits a linear dependence only during the cooling cycle. From the slope of the linear region in the high-temperature range, the activation energy was determined to be 62.8 kJ mol^−1^. Based on the TG analysis results, and the result obtained by heating the[MoO_2_(L)(MeOH)] complex to 200 °C and analysing left-over, it can be assumed that the [MoO_2_(L)(MeOH)] undergoes a thermally induced transformation during heating, resulting in the formation of the polynuclear species [MoO_2_(L)]_*n*_. The activation energy for the transformed [MoO_2_(L)(MeOH)] sample is comparable to that of the polynuclear complex [MoO_2_(L)]_*n*_ (62.8 *vs.* 60.3 kJ mol^−1^), see Table 5, further supporting the conclusion that the heating-induced transformation leads to the formation of the same polynuclear structure. Slightly lower activation energy observed for the polynuclear complex could be attributed to the delocalization of π electrons along the entire polynuclear structure.

The calculated activation energies for the cooling cycles of [MoO_2_(L)]_*n*_ and [MoO_2_(L)(MeOH)] fall within the range consistent with the values reported for semiconducting materials with dominant electronic conductivity. For example, polynuclear and mononuclear Mo(vi) and Cu(ii) complexes with hydrazone ligands (H_2_L^1^, H_2_L^2^) derived from 2-hydroxy-5-nitrobenzaldehyde and 2-hydroxybenzhydrazide or 4-hydroxybenzhydrazide, respectively, whereby in the mononuclear complexes H_2_O or methanol molecule is coordinated at the sixth coordination site of the Mo(vi) center.^38^ Obtained similar *E*_DC_ values for [MoO_2_(L^1^)] and the thermally transformed [MoO_2_(L^1^)(MeOH)] and [MoO_2_(L^2^)(MeOH)] complexes in the cooling cycle (67.2 *vs.* 65.5–66.0 kJ mol^−1^), suggesting that both methanol-containing complexes convert into polynuclear forms upon heating.^38^

A comparable transformation was observed in this study for the polynuclear [MoO_2_(L)]_*n*_ and the mononuclear methanol complex [MoO_2_(L)(MeOH)]. Both compounds exhibit semiconducting behaviour consistent with electronic transport.

The combination of moderate activation energies and the d^0^ electronic configuration of Mo(vi) centres suggest that charge carriers are generated through ligand-to-metal charge transfer (LMCT)^40^ within the π-conjugated thiophene–hydrazone backbone. Once formed, these carriers may migrate by π-electron delocalisation along the ligand framework and possibly *via* Mo=O⋯Mo bridge-assisted hopping between neighbouring units. Comparable O, N-donor coordination environments and *cis*-dioxo Mo(vi) structural motifs have been reported for related Schiff-base systems,^41^ supporting the structural analogy that underlies the proposed transport pathway. Together, these features provide a possible more detailed explanation for the semiconducting response observed in the present materials.

In addition to the electrical conductivity, the dielectric permittivity of the Mo(vi) complexes was determined from the ss-IS measurements. The dielectric properties was analyzed in terms of complex permittivity, ε*(*ω*), as defined:^42^

**Fig. 9:**
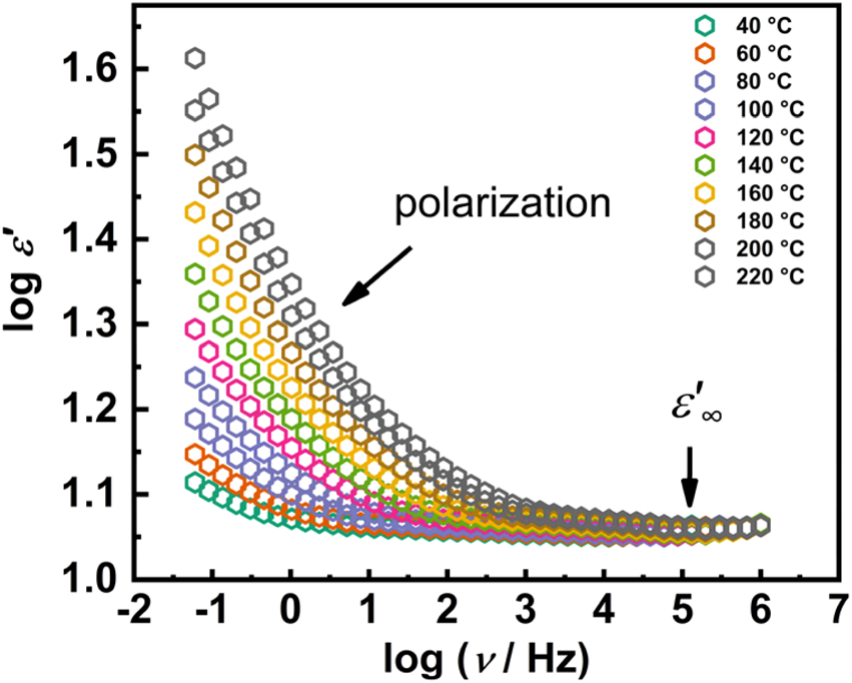
Frequency dependence of real part of the complex permittivity (*ε*′) at different temperatures for [MoO_2_(L)]_*n*_ in double log-scale.

2 ε*(*ω*) = 1/(*iωC*_0_Z*) = *ε*′(*ω*) − *iε*″(*ω*)

where *ε*′(*ω*) and *ε*″(*ω*) are the real and imaginary parts of the complex permittivity. The permittivity spectra, *i.e.* frequency dependence of *ε*′(*ω*), for the sample [MoO_2_(L)]_*n*_ is shown in Fig. 9. The real component, *ε*′(*ω*), is commonly referred to as the dielectric constant.

The dielectric permittivity and its frequency dependence reveal two distinct characteristics, stemming from intrinsic polarisation and bulk permittivity, as shown in Fig. 9. In general, polarization processes are prime contributors to the dielectric response of a material.^42–45^ Under an external electric field, various polarization mechanisms such as atomic, electronic, orientational, and space charge polarization, each characterized by its own relaxation time, contribute to overall dielectric permittivity. At lowest temperatures and high frequencies *ε*′(*ω*) reaches a constant value, as fast polarization processes occur within the sample under the field. Slower processes, such as orientational and space-charge polarization, cannot follow the rapidly changing field and therefore contribute negligibly. On the other hand, as the frequency decreases, *ε*′(*ω*) increases due to enhanced dielectric polarization, dominated by interfacial or space-charge effects. This arises from the accumulation of charge carriers at interfaces in response to an external field.

This type of behavior can be described by the Maxwell–Wagner polarization.^46^ This interfacial mechanism occurs in heterogeneous dielectric systems, where charge carriers accumulate at the interfaces between regions of different conductivity and permittivity between different materials or within different regions of the same material. When these carriers reach the interfacial layer, they accumulate, leading to interfacial polarization. As the frequency increases, charge carriers cannot keep up with field reversals and consequently, the real component of permittivity decreases, eventually stabilizing at a constant high-frequency value corresponding to the dielectric constant, 
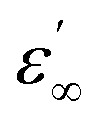
. Similar trends have been reported for other TM complexes, oxide systems, and polymer-based composites, where Maxwell–Wagner polarization plays a crucial role in determining the overall dielectric response.^47–50^

Table 6 presents the values of the dielectric constant for both samples at 40 °C during both the heating and cooling cycles. The values of the dielectric constant are very similar in both the heating and cooling cycles for the [MoO_2_(L)(MeOH)]. The dielectric constant values are ∼11–13. The polynuclear and mononuclear complexes with coordinated methanol exhibit nearly close dielectric constant values.

**Table 6:** The values of dielectric constant at 40 °C and high-frequency for heating–cooling cycles

Sample	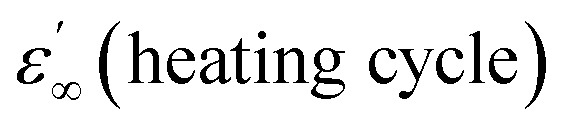	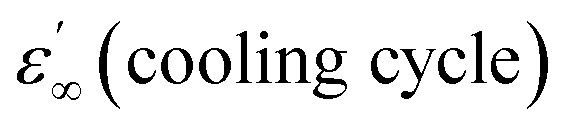
[MoO_2_(L)(MeOH)]	12.73	12.34
[MoO_2_(L)]_*n*_	11.23	11.15

It can be concluded that electrical conductivity is more sensitive to complex and temperature-induced structural changes, making it possible to establish a correlation. In contrast, such a correlation is not observed for the dielectric constant.

## 3 Experimental

### 3.1 Materials and methods

#### 3.1.1. Materials

All the chemicals were commercially available and used without purification, see Table 7.

**Table 7:** The chemicals used in this study

Chemicals	Producer, purity
Salicylaldehyde (C_7_H_6_O_2_)	Fluka AG, >99%, puriss
Thiophene-2-carbohydrazide (C_5_H_6_N_2_OS)	BLDpharm, 99.71%
Ammonium heptamolybdate tetrahydrate ((NH_4_)_6_Mo_7_O_24_·4H_2_O)	Lach – Ner, s.r.o, min. 99.0%
Pentane-2,4-dion (C_5_H_8_O_2_)	Fluka, ≥98%, purum
Nitric acid (*w*(HNO_3_) = 10%)	GRAM-MOL, 65%, p.a.
Methanol (CH_3_OH)	GRAM-MOL, min. 99.5%, p.a.
Acetonitrile (MeCN)	CARLO ERBA Reagents, p.a.
Acetone (C_3_H_6_O)	GRAM-MOL, p.a.
Dichloromethane (CH_2_Cl_2_)	T.T.T., p.a.
Hydrogen peroxide *w*(H_2_O_2_, aq) = 30%	GRAM-MOL, p.a.
Diethyl-ether (C_4_H_10_O)	T.T.T., p.a.
biphenyl (C_12_H_10_)	Sigma Aldrich
Benzyl alcohol (C_7_H_8_O)	TCI, >99.0%

#### 3.1.2. Methods

IR-ATR spectroscopic analysis was used to characterize all synthesized compounds. The method was carried out using a PerkinElmer Spectrum Two FT-IR spectrometer equipped with a diamond UATR accessory. The samples were placed on the diamond reflection element, and the spectrum of each sample was recorded in the wavenumber range from 4000 cm^−1^ to 400 cm^−1^.

Thermogravimetric analysis (TGA) of the samples was performed using a Mettler Toledo TGA/SDTA851e instrument. Measurements were conducted in aluminum oxide (Al_2_O_3_) crucibles, under an oxygen flow, in the temperature range of 25 to 600 °C, with a heating rate of 10 °C min^−1^. The thermograms were processed using Mettler Toledo STARe Evaluation Software 18.00.

Differential scanning calorimetry (DSC) was performed using a Mettler Toledo 823e instrument. The measurement on the H_2_L hydrazone ligand sample was carried out under a nitrogen atmosphere, using an aluminum crucible in the temperature range of 25 to 300 °C, with a heating rate of 10 °C min^−1^. The results were processed using Mettler Toledo STARe Evaluation Software 18.00.

Liquid-assisted grinding (LAG) of the samples was conducted using a Retsch MM200 mill with Teflon (12.5 mL) grinding jars, each containing one Teflon (*d* = 8 mm). Grinding was performed at 25 Hz for one hour. The solvents used were methanol, acetonitrile, and dichloromethane.

One-dimensional nuclear magnetic resonance (NMR) spectra (^1^H and ^13^C) for ligand and [MoO_2_(L)(MeOH)] complex were recorded on a Bruker Avance III HD 400 MHz/54 mm Ascend spectrometer equipped with a 5 mm PA BBI 1H/D BB Z-GRAD probe (SI, Fig. S12–S15). All measurements were performed at 298 K using standard Bruker pulse programs. DMSO-d6 was used as the solvent, and tetramethylsilane (TMS) served as the internal reference for chemical shifts of both proton and carbon nuclei, expressed in parts per million (ppm). [MoO_2_(L)(MeOH)] was selected as a representative complex since it is known that DMSO will coordinate to the metal centre.

The absorption spectra were recorded using a Specord 200 spectrometer manufactured by Analytik Jena (Germany). The wavelength range was 200–600 nm, with a slit width set to 2 nm and a scanning speed of 10 nm s^−1^. Conventional quartz cuvettes (*l* = 1 cm) were used. Solid-state Impedance spectroscopy (ss-IS) was employed to investigate the electrical/dielectric properties of [MoO_2_(L)]_*n*_ and [MoO_2_(L)(MeOH)] complexes. Gold was applied to the pressed samples using a Sputter Coater SC7620. Measurements were carried out using a Novocontrol Alpha-AN dielectric spectrometer^51^ in wide frequency (0.04 Hz–1 MHz) and temperature range (30–220 °C). Experimental Impedance data were analyzed by modelling with an electrical equivalent circuit (EEC) using the complex non-linear least square fitting procedure (CNLSQ), performed with the commercial software ZView.^52^ Gas chromatography analyses were conducted using an Agilent 8860 gas chromatograph (Agilent Technologies) equipped with a flame ionization detector (FID) and an HP-5 capillary column (30 m × 0.32 mm × 0.25 µm), employing helium as the carrier gas. The temperatures of the injection port and detector were held at 250 °C and 200 °C, respectively. Quantification was performed through calibration curves established from authentic standards of all pertinent compounds. The conversion of benzyl alcohol and the formation of aldehyde were measured relative to a biphenyl internal standard, with calibration curves demonstrating exceptional linearity (*r*^2^ = 0.999). Each catalytic run was performed twice to ensure reproducibility of the reported catalytic parameters.

Single crystals of H_2_L, [MoO_2_(L)(MeOH)], and [MoO_2_(L)(H_2_O)]·(CH_3_)_2_CO of suitable quality were selected for X-ray diffraction studies. Data collection was performed on a Rigaku XtaLAB Synergy-S diffractometer equipped with a Dualflex source using Cu Kα radiation (*λ* = 1.54184 Å) and a HyPix detector. Diffraction data were acquired using ω-scan techniques at 293 K for ligand H_2_L^1^, at 100 K for compound [MoO_2_(L)(MeOH)] and 298 K for compound [MoO_2_(L)(H_2_O)]·(CH_3_)_2_CO. Given the satisfactory refinement parameters and overall crystallographic quality of both structures measured at room temperature, we considered the datasets fully reliable and therefore did not repeat all the measurements at 100 K. The data were processed with the CrysAlisPro software package.^53^ Initial structure solution was achieved using dual-space methods implemented in SHELXT,^54^ followed by full-matrix least-squares refinement on *F*^2^ using SHELXL.^55^ Refinement included anisotropic displacement parameters for all non-hydrogen atoms. Hydrogen atoms bonded to carbon were positioned geometrically and refined using a riding model, with *U*_iso_ values set to 1.2*U*_eq_ for CH and CH_2_ groups and 1.5*U*_eq_ for methyl groups. Hydrogen atoms bound to heteroatoms were located from difference Fourier maps during the final refinement stages. All SHELX operations were carried out within the Olex2 crystallographic interface.^56^ Geometric calculations were performed using PLATON,^57^ and molecular graphics were generated with Mercury.^58^ A summary of crystallographic data is provided in Tables S1–S3 and Fig. S6–S8 (SI). Crystallographic data have been deposited with the Cambridge Crystallographic Data Centre under deposition numbers CCDC 2489401–2489403.

#### 3.1.3. Preparation of starting compounds

##### 3.1.3.1. Synthesis of the ligand (H_2_L) by LAG method and in solution

The starting aldehyde and hydrazide (0.3171 mmol) were placed in a Teflon milling jar with a Teflon ball. MeOH, 20 µL, was added, and the mixture was milled at 25 Hz for 60 minutes. The reaction afforded powdered product.

In a 100 mL single-neck round-bottom, 0.50 g (3.52 mmol) of white powder thiophene-2-carbohydrazide (C_5_H_6_N_2_OS) was dissolved in 30 mL of methanol with heating. To the colorless solution, 2-hydroxybenzaldehyde (C_7_H_6_O_2_; *V* = 0.37 mL, *ρ* = 1.166 g mL^−1^, *n* = 3.52 mmol) was added. Upon addition of 2-hydroxybenzaldehyde a pale-yellow transparent solution was formed. The resulting solution was refluxed for two hours. After few days yellow crystals were formed that were filtered.

H_2_L: pale yellow needles, *m* = 0.51 g, yield = 81.53%.

IR-ATR *ν*_max_/cm^−1^: 3400–2600 (O–H); 3257 (–NH); 3067 (=Car–H); 1656 (–C=O); 1606 (–C=N_imine_); 1490 (–C=Car); 1250 (–C–O_phenol_); 648 (C–S).

Found: C, 58.11; H, 3.92, N, 11.12; C_12_H_10_N_2_O_2_S requires C, 58.52; H, 4.09; N, 11.37%.

DSC: *m* = 7.40 mg; *n* = 0.0301 mmol, Δ*H* = 352.8 mJ/0.0301 mmol ≈ 11.8 kJ mol^−1^.

^1^H NMR (DMSO) *δ*/ppm: 6.94 (2H, m, Ar–H), 7.25 (1H, t, thiophene-H), 7.30 (1H, t, Ar–H), 7.57 (1H, d, Ar–H), 7.95 (1H, d, thiphene-H), 8.45 (1H, s, thiophene-H), 8.63 (1H, s, thiophene-H), 8.63 (1H, s, –CH=N), 11.11 (1H, s, –OH), 11.74 (1H, s, –NH).

^13^C NMR (DMSO) *δ*/ppm: 116.8, 119.2, 120.1, 126.5, 127.1, 128.6, 129.6, 131.7, 132.6, 141.2, 148.1, 157.8.

##### 3.1.3.2. Synthesis of [MoO_2_(C_5_H_7_O_2_)_2_]

The complex was prepared according to a known protocol.^59,60^

A yellow powder was isolated: [MoO_2_(C_5_H_7_O_2_)_2_] (*m* = 2.16 g, yield = 38.96%).

#### 3.1.4. Preparation of molybdenum complexes

##### 3.1.4.1. Mechanochemical synthesis

Ligand and starting Mo compound (0.3171 mmol) were placed in a Teflon milling jar with a Teflon ball. Solvent (MeOH, or MeCN or DCM) (20 µL) was added, and the mixture was milled at 25 Hz for 60 minutes. The reaction afforded powdered product. LAG with MeOH provided [MoO_2_(L)(MeOH)] complex, while with MeCN and DCM [MoO_2_(L)]_*n*_ was obtained.

###### 3.1.4.1.1 [MoO_2_(L)(MeOH)]

IR-ATR *ν*_max_/cm^−1^: 3400–2600 (O–H); 3097 (=C_ar_–H); 1597 (–C=N_imin_); 1493 (–C=C_ar_); 1271 (–C–O_phenol_); 1010 (MeOH); 930 and 910 (MoO_2_), 639 (C–S).

Found: C, 38.11; H, 2.04; N, 6.43; C_13_H_12_MoN_2_O_5_S requires C, 38.62; H, 2.99; N, 6.93%.

TGA: *w*_t_ (MeOH, [MoO_2_(L)(MeOH)]) = 7.92%, *w*_exp_ (MeOH, [MoO_2_(L)(MeOH)]) = 8.84%; *w*_t_ (MoO_3_, [MoO_2_(L)(MeOH)]) = 35.61%, *w*_exp_ (MoO_3_, [MoO_2_(L)(MeOH)]) = 33.57%.

^1^H NMR (DMSO) *δ*/ppm: 6.96 (1H, d, Ar–H), 7.09 (1H, td, thiophene-H), 7.22 (1H, m, Ar–H), 7.53 (1H, m, Ar–H), 7.72 (1H, m, Ar–H), 7.87 (2H, dd, thiophene-H), 8.92 (1H, s, –CH=N).

^13^C NMR (DMSO) *δ*/ppm: 118.9, 120.8, 122.2, 128.9, 131.8, 132.6, 133.3, 134.6, 135.3, 156.7, 159.7.

###### 3.1.4.1.2 [MoO_2_(L)]_*n*_

IR-ATR *ν*_max_/cm^−1^: 3029 (=C_ar_–H); 1600 (–C=N_imin_); 1445 (–C=C_ar_); 1268 (–C–O_fenol_); 925 (Mo=O); 850 (Mo=O⋯Mo=O), 640 (C–S).

Found: C, 38.14; H, 2.02; N, 7.11; C_12_H_8_MoN_2_O_4_S requires C, 38.72.2; H, 2.17; N, 7.53%.

TGA: *w*_t_ (MoO_3_, [MoO_2_(L)]_*n*_) = 38.67%, *w*_exp_ (MoO_3_, [MoO_2_(L)]_*n*_) = 36.82%.

##### 3.1.4.2. Solution-based synthesis

###### 3.1.4.2.1 Mononuclear complexes [MoO_2_(L)(D)] D = MeOH or H_2_O

In a one-neck round-bottom flask, 37.9 mg (0.15 mmol) of the ligand H_2_L is dissolved in 20 mL of methanol or acetonitrile with heating. 0.05 g (0.15 mmol) of the yellow powder [MoO_2_(C_5_H_7_O_2_)_2_] is added. The solution is refluxed for two hours.

From MeOH [MoO_2_(L)(MeOH)]: orange needles (*m* = 41.8 mg, *η* = 68%).

From acetonitrile [MoO_2_(L)(H_2_O)]: orange product (*m* = 4.7 mg, *η* = 7.86%).

IR-ATR *ν*_max_/cm^−1^: 3600–2800 (O–H); 3093 (=C_ar_–H); 1653 (H_2_O); 1598 (–C=N_imin_); 1493 (–C=C_ar_); 1268 (–C–O_phenol_); 940 and 910 (MoO_2_), 638 (C–S).

Found: C, 39.40; H, 2.46; N, 7.09; C_12_H_10_MoN_2_O_5_S requires C, 36.94; H, 2.58, N, 7.18%.

TGA: *w*_t_ (MeOH, [MoO_2_(L)(H_2_O)]) = 4.79%, *w*_exp_ (H_2_O, [MoO_2_(L)(H_2_O)]) = 4.62%; *w*_t_ (MoO_3_, [MoO_2_(L)(H_2_O)]) = 36.89%, *w*_exp_ (MoO_3_, [MoO_2_(L)(H_2_O)]) = 35.69%.

###### 3.1.4.2.2 Polynuclear complexes [MoO_2_(L)]_*n*_

In a one-neck round-bottom flask, 37.9 mg (0.15 mmol) of the ligand H_2_L is dissolved in 20 mL of dichloromethane with heating. 0.05 g (0.15 mmol) of the yellow powder [MoO_2_(C_5_H_7_O_2_)_2_] is added. The solution is refluxed for two hours.

[MoO_2_(L)]_*n*_: red precipitate (*m* = 11.6 mg, *η* = 20.28%).

##### 3.1.4.3. *In situ* synthesis

*In situ* synthesis from MeOH provided [MoO_2_(L)(MeOH)] complex, while from MeCN and DCM [MoO_2_(L)]_*n*_ was obtained.

## 4 Conclusions

The mechanochemical synthesis of molybdenum complexes derived from 2-thiophenecarboxylic hydrazone afforded materials that combine catalytic and semiconducting properties while adhering to green chemistry principles. The solvent-free mechanochemical route eliminates hazardous solvents, offering a sustainable alternative to conventional solution-based synthesis. Catalytic evaluation in the benchmark oxidation of benzyl alcohol, conducted under mild conditions with aqueous hydrogen peroxide as a clean oxidant, revealed reproducible activity for both the mononuclear [MoO_2_(L)(MeOH)] and the polynuclear [MoO_2_(L)]_*n*_ complexes, with conversions of 8–13% and aldehyde selectivity consistently exceeding 70%. Solid-state impedance spectroscopy revealed semiconducting properties, with DC conductivities on the order of ∼10^−12^ (Ω cm)^−1^ @200 °C and activation energies derived from cooling cycles are in the range ∼60–63 kJ mol^−1^ which is consistent with values reported for semiconducting systems dominated by electronic conductivity. Dielectric permittivity spectra confirmed Maxwell–Wagner-type interfacial polarization, with dielectric constants of ∼11–13. These findings demonstrate that mechanochemically synthesized molybdenum complexes represent sustainable bifunctional systems with selective catalytic and electronic behavior. Nevertheless, further optimization of the catalytic conditions and/or structural modifications of the complexes are required to enhance their catalytic efficiency.

## Author contributions

Conceptualization: L. P., J. P. Data curation: L. P., F. M., J. S., M. R., J. P. Formal analysis: F. M.; J. S., M. R. Funding acquisition: L. P., J. P. Investigation: L. P., J. S., M. R., J. P. Methodology: L. P., J. P. Project administration: L. P., J. P. Resources: L. P., J. P. Software: L. P., J. S., M. R., J. P. Supervision: L. P., J. P. Validation: F. M.; J. S., M. R. Visualization: L. P.; J. S., M. R., J. P. Writing – original draft: L. P., J. S., J. P. Writing – review & editing: L. P., J. S., M. R., J. P.

## Conflicts of interest

There are no conflicts to declare.

## Supplementary Material

RA-015-D5RA07456H-s001

RA-015-D5RA07456H-s002

## Data Availability

CCDC 2489401–2489403 contain the supplementary crystallographic data for this paper.^61*a*–*c*^ The data supporting this article have been included as part of the supplementary information (SI). Supplementary information: IR-ATR, NMR, UV-Vis spectra of ligand and obtained complexes, DSC of ligand, crystallographic data for ligand and complexes. See DOI: https://doi.org/10.1039/d5ra07456h.
