# Supplementary material for: From structure to function: tunable electrical and catalytic properties in rare Mo(vi)-thiophene-2-carboxylic acid hydrazone complexes obtained mechanochemically
Source: RSC Adv. 2025 Nov 26;15(54):46194–206. doi: 10.1039/d5ra07456h (PMC12650264; doi:10.1039/d5ra07456h)
Supplement: RA-015-D5RA07456H-s001 [file RA-015-D5RA07456H-s001.pdf]

Supplementary Information (SI) for RSC Advances.
This journal is © The Royal Society of Chemistry 2025

From Structure to Function: Tunable Electrical and Catalytic Properties in Rare Mo(VI)–Thiophene-2-Carboxylic Acid Hydrazone Complexes

Luka Pavić, Filip Miočić, Marta Razum, Josipa Sarjanović and Jana Pisk

Table of Contents

Figure S1. DSC curve for the ligand H_2L 2

Figure S2. IR–ATR spectrum for the ligand H_2L 2

Figure S3. IR–ATR spectrum for the mononuclear complex $[MoO_2(L)(MeOH)]$ 3

Figure S4. IR–ATR spectrum for the mononuclear complex $[MoO_2(L)(H_2O)]$ 3

Figure S5. IR–ATR spectrum for the polynuclear complex $[MoO_2(L)]_n$ 4

Table S1. Experimental and crystallographic data for crystal structure determined in this work. 5

Table S2. Selected bond lengths in the determined crystal structures in this work. 6

Table S3. Hydrogen bond parameters in the determined crystal structures in this work. 7

Figure S6. (a) Molecular structure of the ligand **H_2L**. Atoms are shown as thermal ellipsoids with 50% probability. (b) Packing of molecules in the unit cell shown along *b*– axis. (c) Packing of molecules in the unit cell shown along *c*– axis. (d) Packing of the ligand **H_2L** in the crystal structure. Hydrogen bonds are highlighted as orange dashed lines. 9

Figure S7. (a) Molecular structure of the mononuclear complex $[MoO_2(L)(MeOH)]$. Atoms are shown as thermal ellipsoids with 50% probability. (b) Packing of molecules in the unit cell shown along *a*– axis. (c) Packing of molecules in the unit cell shown along *b*– axis. (d) Packing of the mononuclear complex $[MoO_2(L)(MeOH)]$ in the crystal structure. Hydrogen bonds are highlighted as orange dashed lines. 11

Figure S8. (a) Molecular structure of the mononuclear complex $[MoO_2(L)(H_2O)]\cdot(CH_3)_2CO$. Atoms are shown as thermal ellipsoids with 50% probability. (b) Packing of molecules in the unit cell shown along *a*– axis. (c) Packing of molecules in the unit cell shown along *b*– axis. (d) Packing of the mononuclear complex $[MoO_2(L)(H_2O)]\cdot(CH_3)_2CO$ in the crystal structure. Hydrogen bonds are highlighted as orange dashed lines. 13

Table S4. Comparison of catalytic parameters reported in the literature. 14

Figure S9. Plausible mechanism for oxidation of benzyl alcohol with H_2O_2 catalyzed by $[MoO_2(L)(MeOH)]$ complex, based on the literature data.ʼ 15

Table S5. UV-Vis data for complex and corresponding complex dissolved in methanol (c=10^{-5} mol dm^{-3}). 15

Figure S10. Spectral changes observed after the successive addition of one drop portion of 30% hydrogen peroxide to $[MoO_2(L)(MeOH)]$ complex in MeOH (orange, grey and yellow curve). The blue curve presents the UV-Vis spectrum of the ligand in MeOH. 16

Figure S11. Complex impedance plane (Nyquist plot) and spectra for $[MoO2(L)]_n$ @200 °C with corresponding EEC used for fitting the data. Symbols and red line represent experimental data, while green line depict the fit obtained through EEC modelling. 17

Figure S12. ^{1}H NMR spectra for the ligand H_2L in DMSO. 17

Figure S13. ^{13}C NMR spectra for the ligand H_2L in DMSO. 18

Figure S14. ^{1}H NMR spectra for the complex $[MoO_2(L)(MeOH)]$ in DMSO. 18

Figure S15. ^{13}C NMR spectra for the complex $[MoO_2(L)(MeOH)]$ in DMSO. 19

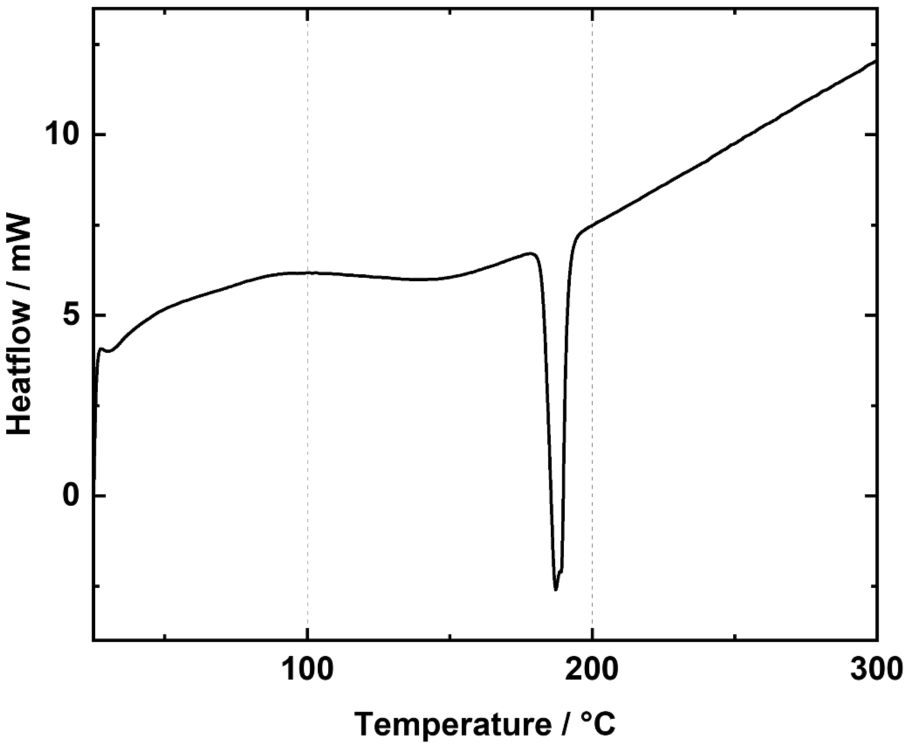

Figure S1. DSC curve for the ligand H_2L.

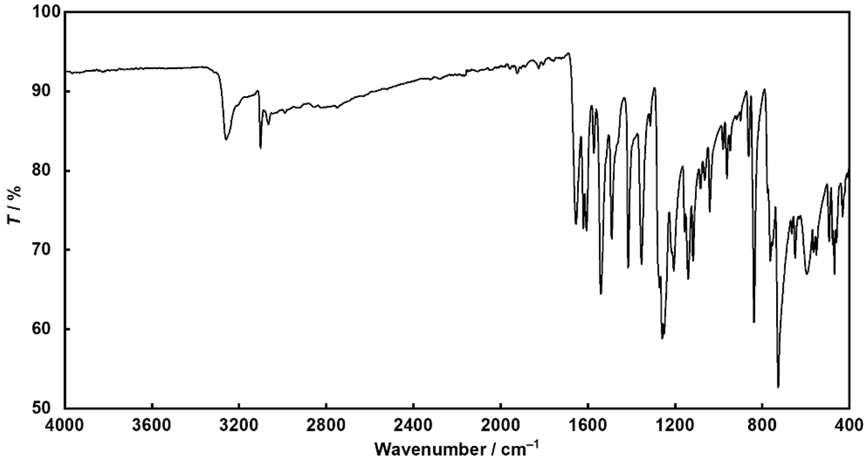

Figure S2. IR–ATR spectrum for the ligand H_2L.

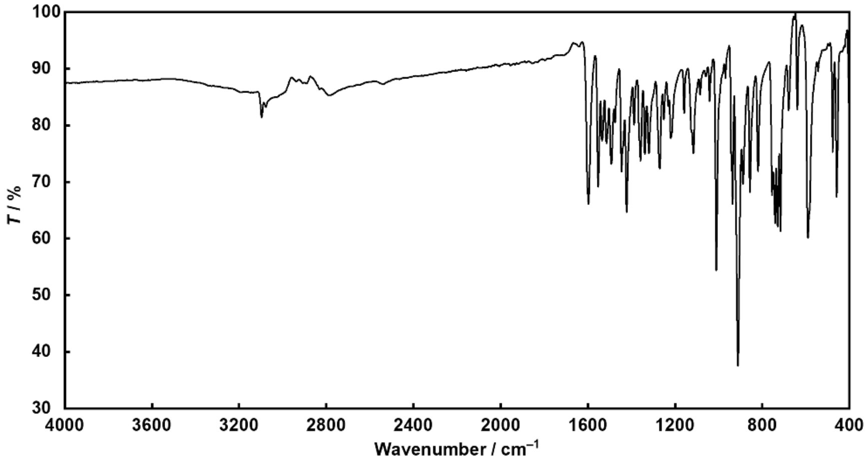

Figure S3. IR–ATR spectrum for the mononuclear complex [MoO_2(L)(MeOH)].

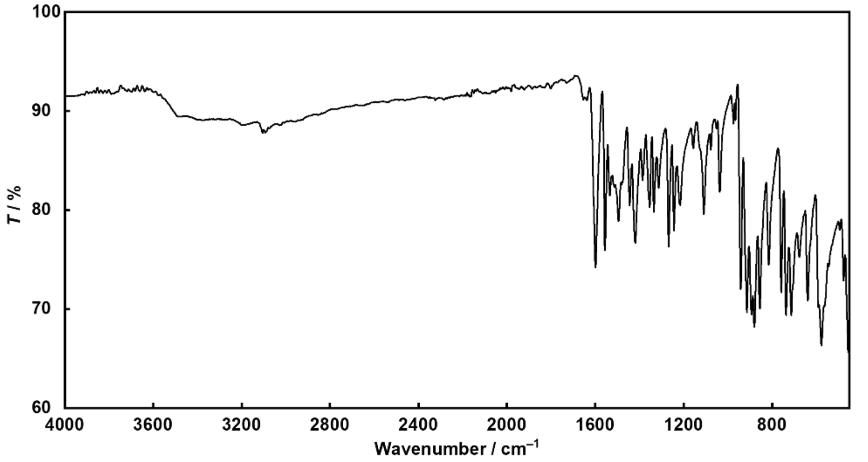

Figure S4. IR–ATR spectrum for the mononuclear complex [MoO_2(L)(H_2O)].

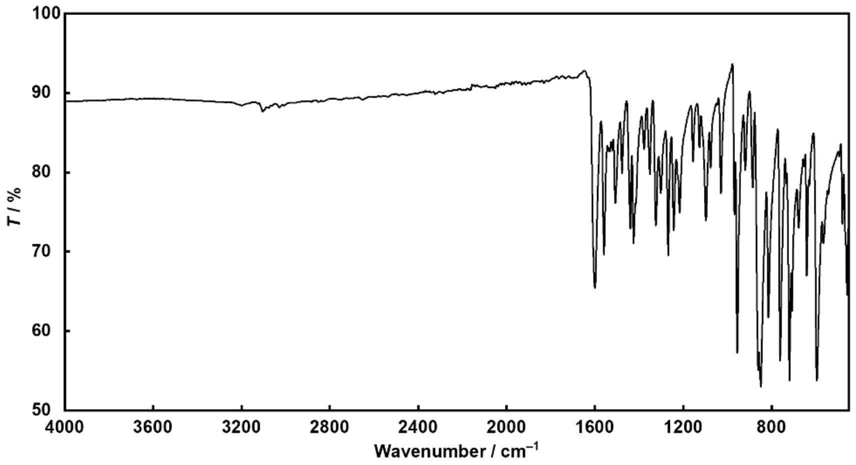

Figure S5. IR–ATR spectrum for the polynuclear complex $[MoO_2(L)]_n$.

Table S1. Experimental and crystallographic data for crystal structure determined in this work**.**

**Identifier**	**H_2L**	**$[MoO_2(L)(MeOH)]$**	**$[MoO_2(L)(H_2O)]\cdot(CH_3)_2CO$**
Empirical formula	$C_{12}H_{10}N_2O_2S$	$C_{13}H_{12}MoN_2O_5S$	$C_{15}H_{16}MoN_2O_6S$
M_r	246.28	404.248	448.30
T/K	293(2)	100.15	298.00
Crystal system	orthorhombic	orthorhombic	monoclinic
Space group	$Pna2_1$	$Pbcn$	$P2_1/c$
a/Å	19.4454(3)	14.8458(2)	7.74290(10)
b/Å	5.36070(10)	7.2875(1)	10.64170(10)
c/Å	10.9087(2)	26.5865(3)	21.2775(4)
α/°	90	90	90
β/°	90	90	91.132(2)
γ/°	90	90	90
V/Å^3	1137.13(3)	2876.36(6)	1752.87(4)
Z	4	8	4
ρ_{calc}/ g cm^{-3}	1.439	1.867	1.699
μ/mm^{-1}	2.466	9.053	7.553
F(000)	512.0	1623.9	904.0
Crystal size/mm^3	0.25×0.15×0.1	0.33×0.04×0.03	0.29×0.05×0.02
Radiation	Cu Kα (λ = 1.54184)		
2Θ range/°	9.096 to 159.492	6.64 to 160.26	8.312 to 159.962
Index ranges	-22 ≤ h ≤ 24,<br>-6 ≤ k ≤ 6,<br>-13 ≤ l ≤ 13	-18 ≤ h ≤ 17,<br>-9 ≤ k ≤ 9,<br>-33 ≤ l ≤ 33	-9 ≤ h ≤ 9,<br>-13 ≤ k ≤ 12,<br>-27 ≤ l ≤ 26
Reflections collected	7899	51987	29131
Independent reflections	2155<br>[R_{int} = 0.0345,<br>R_{sigma} = 0.0304]	3112<br>[R_{int} = 0.0493,<br>R_{sigma} = 0.0190]	3761<br>[R_{int} = 0.0515,<br>R_{sigma} = 0.0297]
Data/restraints/parameters	2155/9/193	3112/18/196	3761/3/236
Goodness-of-fit on F^2, S[a]	1.060	1.039	1.087
Final R and wR[b] values [I≥2σ (I)]	R_1 = 0.0277,<br>wR_2 = 0.0736	R_1 = 0.0361,<br>wR_2 = 0.0911	R_1 = 0.0365,<br>wR_2 = 0.0910
Final R and wR[b] values [all data]	R_1 = 0.0293,<br>wR_2 = 0.0747	R_1 = 0.0368,<br>wR_2 = 0.0917	R_1 = 0.0365,<br>wR_2 = 0.0910
Largest diff. peak/hole / e Å^{-3}	0.12/-0.10	1.65/-0.98	0.41/-0.67

$^{a}S = \{\sum[w(F_o^2 - F_c^2)2]/(N_r - N_p)\}^{1/2}$ where N_r=number of independent reflections, $N_p =$ number of refined parameters.

$^{b}R = \sum||F_o| - |F_c|/\sum|F_o||;\ wR = \{\sum[w(F_o^2 - F_c^2)2]/\sum[w(F_o^2)2]\}^{1/2}$

Table S2. Selected bond lengths in the determined crystal structures in this work.

**H_2L**			
Atoms	Bond length/Å	Atoms	Bond length/Å
S2B–C11A	1.690(9)	C3–C4	1.380(4)
S2B–C9	1.705(3)	C6–C5	1.373(3)
C1–C7	1.454(3)	C4–C5	1.388(4)
C1–C2	1.407(3)	C10B–C11B	1.393(14)
C1–C6	1.395(3)	C10B–C9	1.343(11)
C7–N2	1.271(3)	C11B–C11A	1.335(9)
C8–N1	1.358(3)	C10A–C12B	1.39(3)
C8–O1	1.212(3)	C10A–C9	1.38(2)
C8–C9	1.479(3)	C12B–C12A	1.34(2)
N1–N2	1.376(2)	C12A–S1A	1.69(3)
O2–C2	1.359(3)	S1A–C9	1.681(11)
C3–C2	1.387(3)		
**$[MoO_2(L)(MeOH)]$**			
Atoms	Bond length/Å	Atoms	Bond length/Å
Mo1–O1	2.021(2)	N2–C7	1.298(4)
Mo1–O2	1.926(2)	C8–C9	1.455(4)
Mo1–O5	2.342(2)	C1–C7	1.439(4)
Mo1–N2	2.229(3)	C1–C2	1.412(5)
Mo1–O3	1.691(3)	C1–C6	1.414(4)
Mo1–O4	1.716(3)	C2–C3	1.396(5)
C10B–C9	1.73(2)	C6–C5	1.377(5)
C10B–C11B	1.646(18)	C9–S1A	1.667(4)
S1B–C9	1.519(6)	C9–C10A	1.654(10)
S1B–C12B	1.409(15)	C3–C4	1.391(5)
O1–C8	1.313(4)	C5–C4	1.390(5)
O2–C2	1.335(4)	C12B–C11B	1.353(15)
O5–C13	1.428(4)	S1A–C12A	1.541(5)
N1–N2	1.402(4)	C12A–C11A	1.349(8)
N1–C8	1.304(4)	C11A–C10A	1.667(12)
**$[MoO_2(L)(H_2O)]\cdot(CH_3)_2CO$**			
Atoms	Bond length/Å	Atoms	Bond length/Å
Mo1–O1	2.0278(18)	C7–C1	1.442(4)
Mo1–O2	1.9189(19)	C1–C6	1.409(4)
Mo1–O5	2.377(2)	C4–C5	1.378(5)
Mo1–O4	1.698(2)	C14–C15	1.492(5)
Mo1–N2	2.238(2)	C14–C13	1.474(5)
Mo1–O3	1.694(2)	C6–C5	1.380(5)
O1–C8	1.318(3)	C9–S1A	1.690(3)
O2–C2	1.346(3)	C9–C10A	1.532(11)
N2–N1	1.394(3)	C9–C10B	1.59(2)
N2–C7	1.285(4)	C9–S1B	1.444(11)
N1–C8	1.298(4)	S1A–C12A	1.703(4)
O6–C14	1.212(4)	C10A–C11A	1.455(10)

Continuation of table S2 from previous page			
C8–C9	1.457(4)	C12A–C11A	1.380(6)
C2–C3	1.394(4)	C10B–C11B	1.50(2)
C2–C1	1.399(4)	C11B–C12B	1.34(2)
C3–C4	1.381(4)	C12B–S1B	1.618(13)

Table S3. Hydrogen bond parameters in the determined crystal structures in this work.

**H_2L**					
D–H···A	D–H	H···A	D···A	∠D–H···A	Symmetry code
N1–H1···S1A	0.86	2.72	3.084(10)	107	Intra
N1–H1···O2	0.86	2.21	3.027(2)	159	3/2–x, ½+y, –½+z
O2–H2···N2	0.82	1.90	2.622(2)	146	Intra
C6–H6···O1	0.93	2.37	3.161(3)	143	3/2–x, –½+y, –½+z
**$[MoO_2(L)(MeOH)]$**					
D–H···A	D–H	H···A	D···A	∠D–H···A	Symmetry code
O5–H5···N1	0.84	1.92	2.760(3)	174	3/2–x, –½+y, z
C5–H5A···O4	0.95	2.55	3.327(5)	139	½+x, ½–y, 1–z
C10A–H12A···O1	0.95	2.43	3.285(9)	150	1–x , ½–y, 1–z
C12A–H12A···O4	0.95	2.31	3.231(6)	163	½+x, ½+y, ½–z
**$[MoO_2(L)(H_2O)]\cdot(CH_3)_2CO$**					
D–H···A	D–H	H···A	D···A	∠D–H···A	Symmetry code
O5–H5A···O6	0.90	1.93	2.827(3)	175	1–x,–y,–z
O5–H5B···N1	0.90	2.03	2.877(3)	157	1–x,1–y,1–z
C7–H7···O6	0.93	2.55	3.414(4)	156	1–x,1–y,1–z
C10A–H10A···O5	0.93	2.58	3.353(11)	140	1–x,1–y,1–z
C11A–H11A···O4	0.93	2.54	3.441(7)	164	x, –1+y,z
C13–H13A···O4	0.96	2.39	3.258(5)	150	1–x,–y,–z

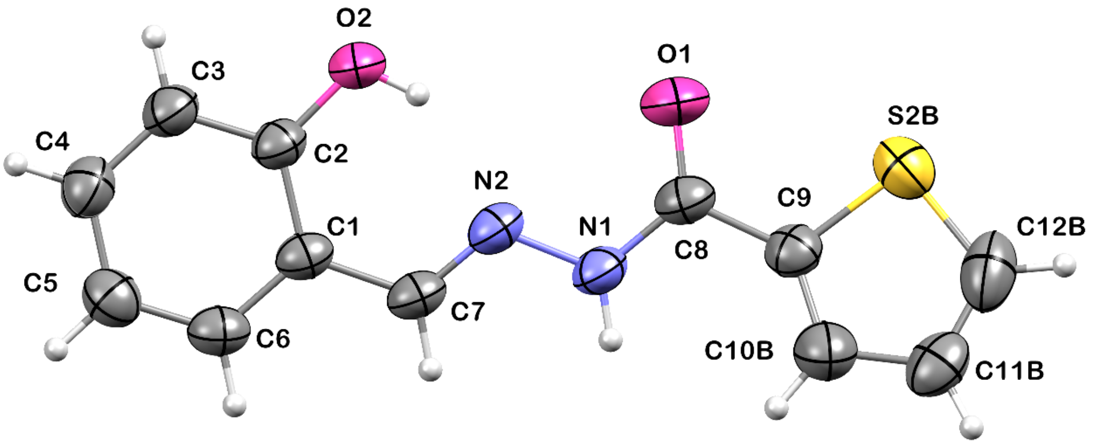

(a)

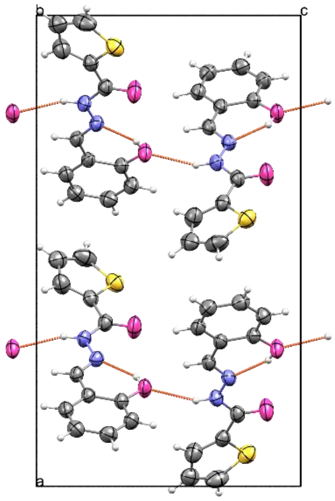

(b)

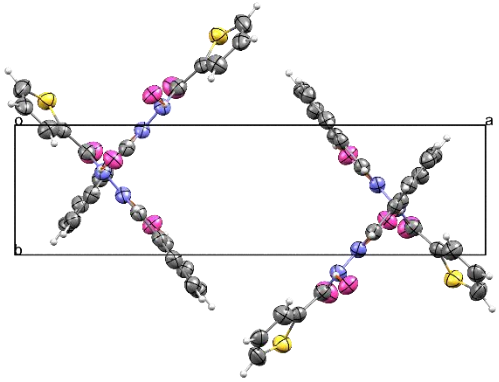

(c)

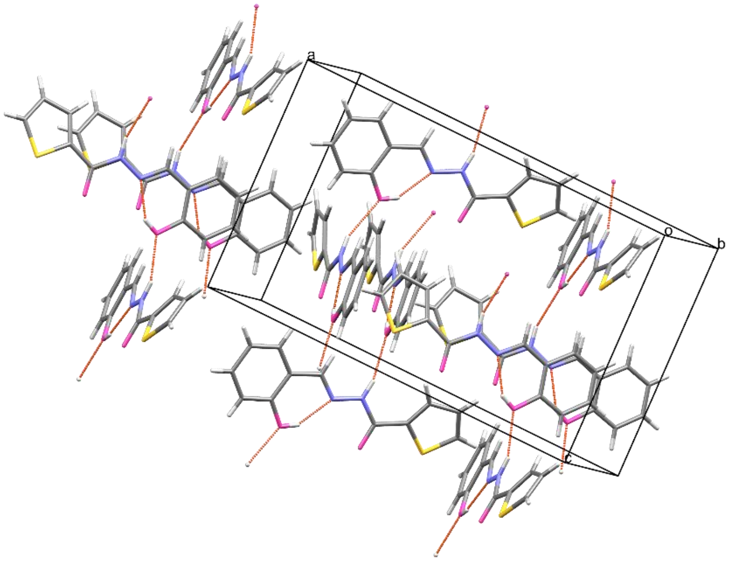

(d)

Figure S6. (a) Molecular structure of the ligand **H_2L**. Atoms are shown as thermal ellipsoids with 50% probability. (b) Packing of molecules in the unit cell shown along *b*– axis. (c) Packing of molecules in the unit cell shown along *c*– axis. (d) Packing of the ligand **H_2L** in the crystal structure. Hydrogen bonds are highlighted as orange dashed lines.

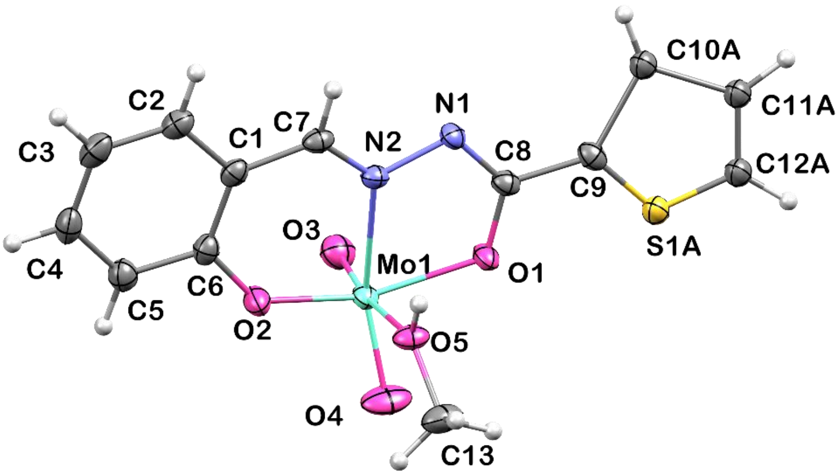
C10A
C2
N1
C11A
C3
C7
C1
N2
C8
C12A
C9
O3
S1A
Mo1
O1
C4
C6
C5
O2
O5
O4
C13

(a)

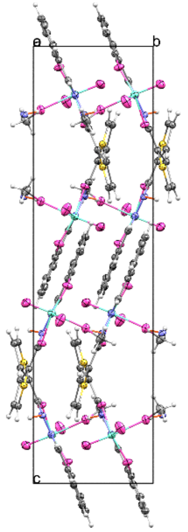

(b)

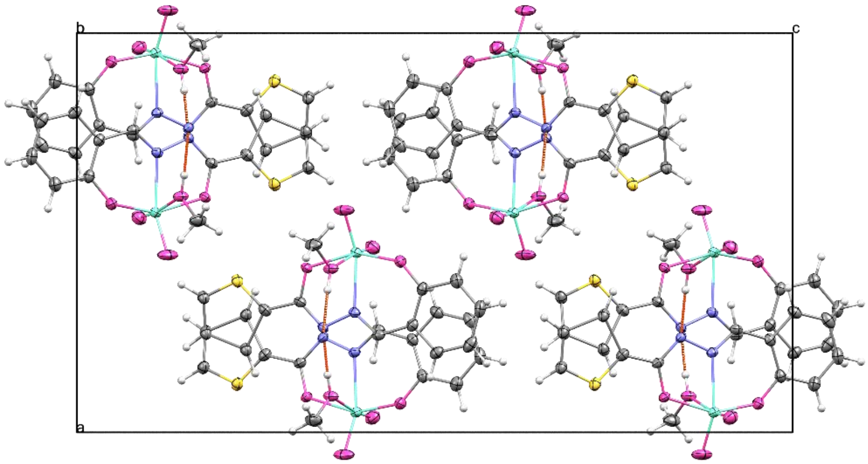

(c)

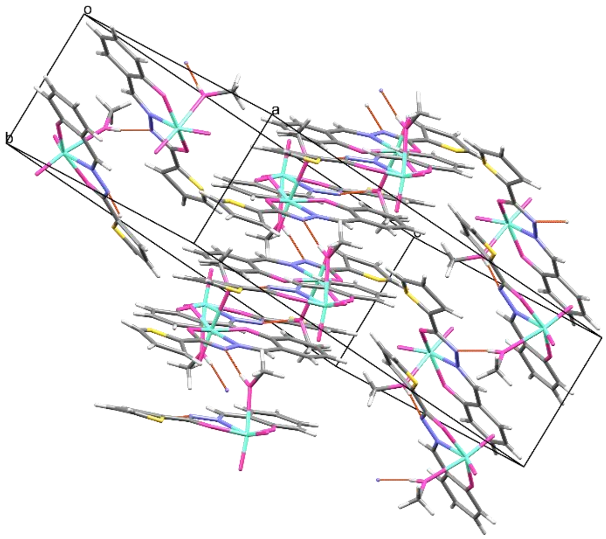

(d)

Figure S7. (a) Molecular structure of the mononuclear complex [MoO_2(L)(MeOH)]. Atoms are shown as thermal ellipsoids with 50% probability. (b) Packing of molecules in the unit cell shown along *a*– axis. (c) Packing of molecules in the unit cell shown along *b*– axis. (d) Packing of the mononuclear complex [MoO_2(L)(MeOH)] in the crystal structure. Hydrogen bonds are highlighted as orange dashed lines.

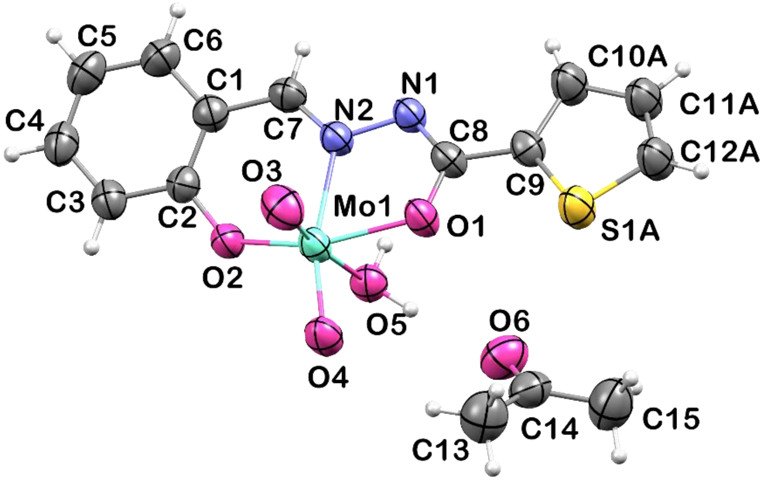
C5
C6
C1
C10A
N1
C11A
C4
C7
N2
C8
C12A
O3
C3
C2
Mo1
C9
O1
S1A
O2
O5
O6
O4
C15
C14
C13

(a)

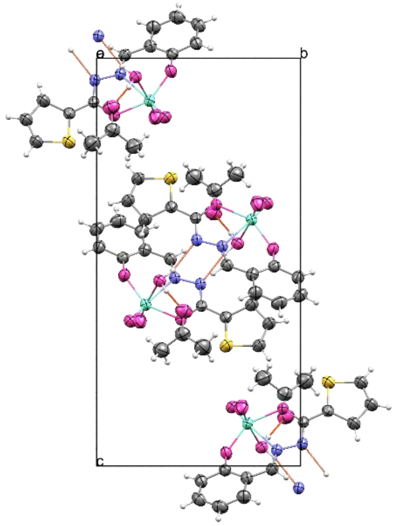

(b)

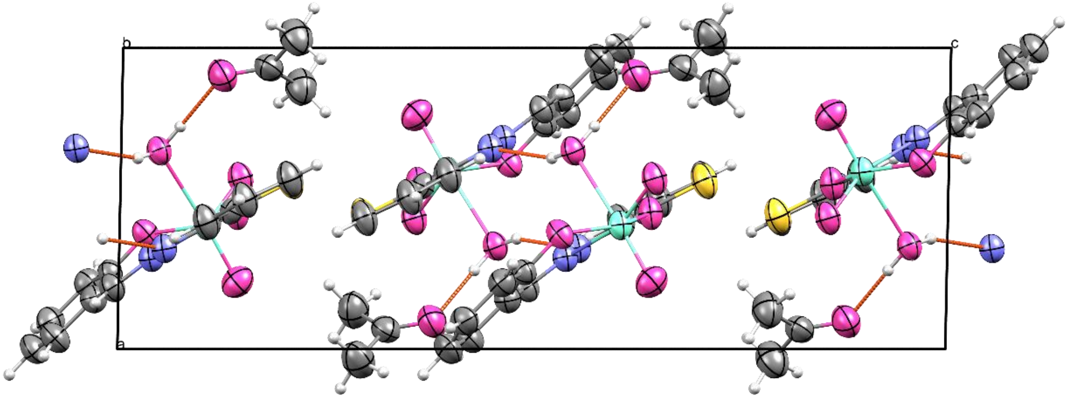

(c)

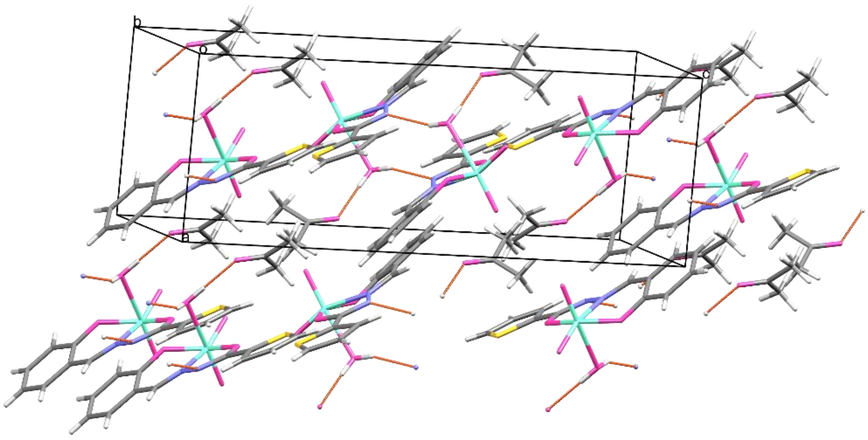

(d)

Figure S8. (a) Molecular structure of the mononuclear complex $[MoO_2(L)(H_2O)]\cdot(CH_3)_2CO$. Atoms are shown as thermal ellipsoids with 50% probability. (b) Packing of molecules in the unit cell shown along *a*– axis. (c) Packing of molecules in the unit cell shown along *b*– axis. (d) Packing of the mononuclear complex $[MoO_2(L)(H_2O)]\cdot(CH_3)_2CO$ in the crystal structure. Hydrogen bonds are highlighted as orange dashed lines.

Table S4. Comparison of catalytic parameters reported in the literature.

**Complex type**	**Ligand**	**Reaction conditions**	**Con**	**Sel/Yield**	**TOF**	**TON**	**Ref**
[MoO$_2$(L)(H$_2$O)]	3-ethoxysalicylaldehyde with 4-aminobenzohydrazide	benzyl alcohol (1 mmol), H2O2, catalyst (0.006 mmol), CH3CN (5 mL), reflux conditions, 2 h.	NR	Yield: 92	76.7	NR	[i]
[MoO$_2$(L)(EtOH)]	Nicotinic hydrazide with 5-nitrosalicylaldehyde	benzyl alcohol (1 mmol), UHP (2 mmol), catalyst (0.006 mmol), CH3CN (10 mL) under reflux conditions, 2h	NR	Yield: 91	76	NR	[ii]
[MoO$_2$(VIH)]$_4$	3-methoxy-2-hydroxybenzaldehyde and isonicotinyl hydrazide	benzyl alcohol (20 mmol), TBHP in water (40 mmol), catalyst (0.1 mmol), 80 °C, 5 h	67	Sel: 45	222	181	[iii]
[MoO$_2$(VIH)(EtOH)]			Low*	NR	Low*	Low*	
[MoO$_2$(SIH)]$_n$	salicylaldehyde and isonicotinic acid hydrazide	benzyl alcohol (20 mmol), H_2O_2 (40 mmol), MeCN (3 mL), catalyst (0.1 mmol), 80 °C, 2 h	11	Sel: 90	23	22	[iv]

NR=not reported

*The corresponding results were not included in the published version owing to suboptimal performance. As we have access to the original dataset, the raw data are provided here for reference: Con: 14%, TON: 27, TOF$_{20\ min}$: 13

[i] H. Kargar and M. Fallah-Mehrjardi*, Inorg. Chem. Res.* 2021, **5**, 201-206.

[ii] H. Kargar, P. Forootan , M. Fallah-Mehrjardi, R. Behjatmanesh-Ardakani , H. A. Rudbari , K. S. Munawar, M. Ashfaq and M. Nawaz, *Inorg. Chim. Acta*, 2021, **523**, 120414.

[iii] E. Topić, J. Sarjanović, D. Musija, M. Mandarić, A. Cocut, T. Hrenar, D. Agustin, J. Pisk and V. Vrdoljak, *Dalton Trans.*, 2025, **54**, 5532–5545.

[iv] E. Topić, J. Sarjanović, D. Musija, M. Mandarić, T. Hrenar, J. Pisk and V. Vrdoljak, *RSC Adv.*, 2025,**15**, 3547-3561

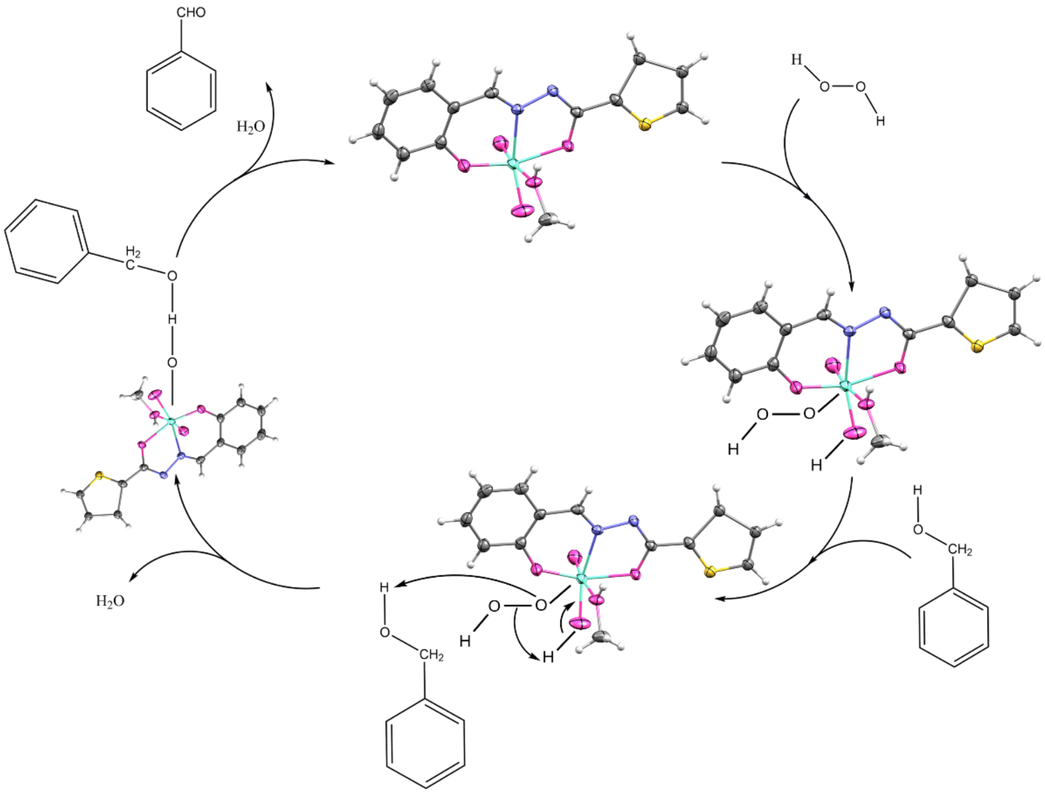

Figure S9. Plausible mechanism for oxidation of benzyl alcohol with H_2O_2 catalyzed by $[MoO_2(L)(MeOH)]$ complex, based on the literature data.[5,6]

Table S5. UV-Vis data for complex and corresponding complex dissolved in methanol (c=10^{-5} mol dm^{-3}).

Compound	λ/nm, A
H_2L	296 (1.046789),<br>306(1.165945)<br>333 (0.949566)
$[MoO_2(L)(MeOH)]$	296 (1.161646)<br>306 (1.297342)<br>332 (1.06628)

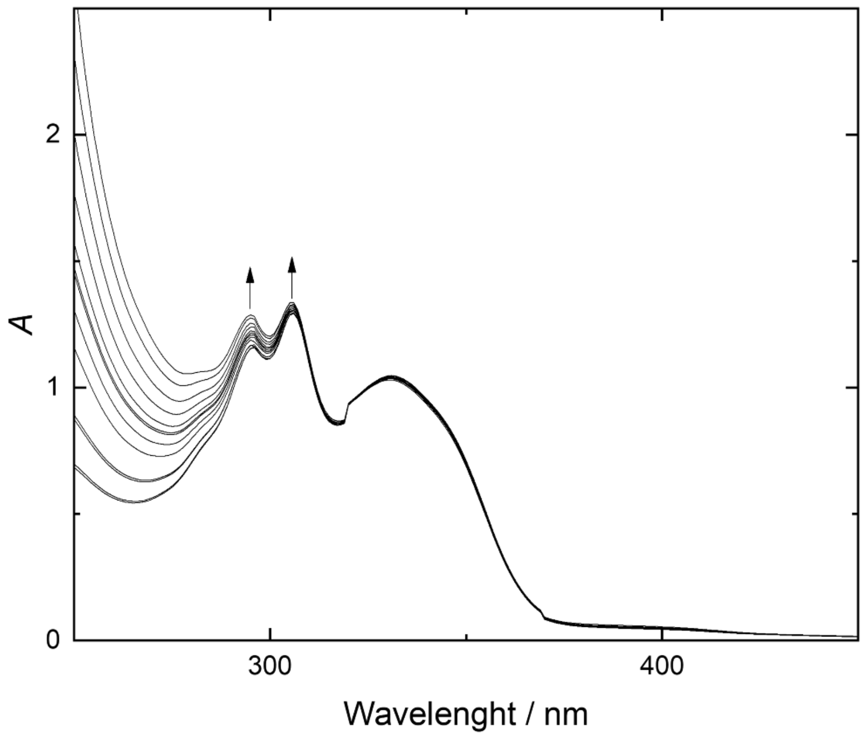

Figure S10. Spectral changes observed after the successive addition of one drop portion of 30% hydrogen peroxide to [MoO_2(L)(MeOH)] complex in MeOH (orange, grey and yellow curve). The blue curve presents the UV-Vis spectrum of the ligand in MeOH.

Based on the reported literature data, we prepared a methanolic solution ($c = 10^{-5}$ mol dm^{-3}) of the [MoO_2(L)(MeOH)] complex, which was titrated with 30% H_2O_2 dissolved in 10 mL of MeOH, and the changes were followed by electronic absorption spectroscopy. The addition of H_2O_2 resulted in an increase of the intensity for the bands at 296 and 307 nm. The increase in intensity upon adding H_2O_2 suggests the formation or accumulation of a new species, likely a peroxo–Mo(VI) species. No changes are observed for the band at 332 nm, implying that this band arises from a stable moiety in the complex that does not participate in peroxide binding or transformation, probably ligand. The similar behaviour of Mo complexes was described previously.[7,8,9]

[5] A. Bovand, H. Kargar and M. Fallah-Mehrjardi, *J. Iran. Chem. Soc.*, 2022, **19**, 3463-3471.

[6] X. Liu, S. Yang, L. Yang, H. Yu, T. Zhang and W. Wang, *Afinidad*, 2023, **80**, 598.

[7] M. R. Maurya, R. Tomar, L. Rana and F. Avecilla, *Eur. J. Inorg. Chem.*, 2018, 2952-2964.

[8] M. R. Maurya, L. Rana and F. Avecilla, Polyhedron, 2017

[9] M. R. Maurya, L. Rana, N. Jangra and F. Avecilla, *ChemistrySelect*, 2017, **2**, 6767-6777.

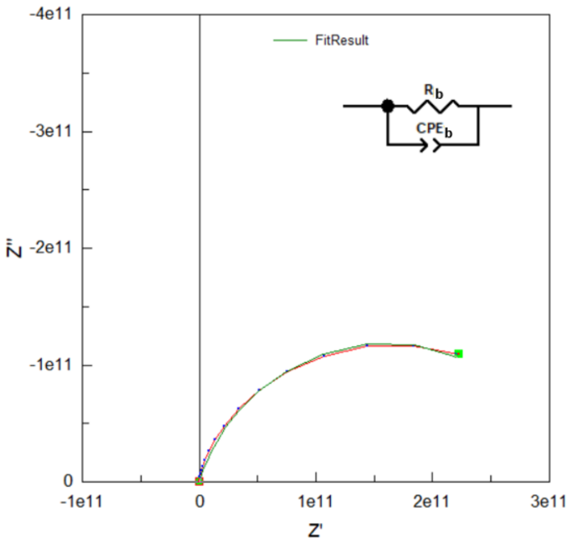

Figure S11. Complex impedance plane (Nyquist plot) and spectra for $[MoO2_{(L)}]_n$ @200 °C with corresponding EEC used for fitting the data. Symbols and red line represent experimental data, while green line depict the fit obtained through EEC modelling.

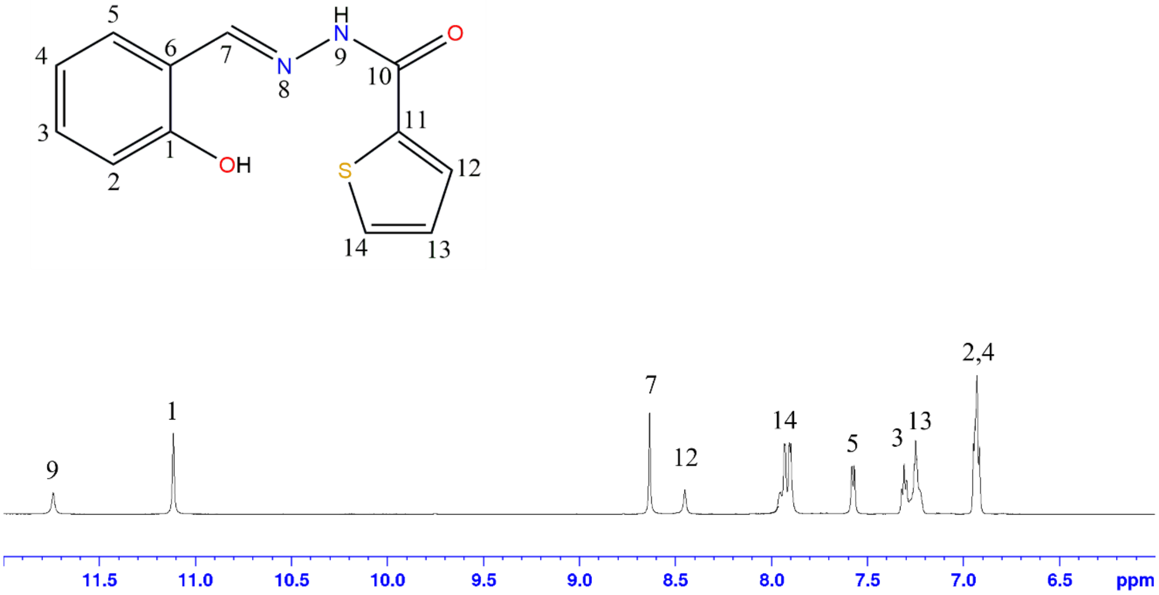

Figure S12. ^{1}H NMR spectra for the ligand H_2L in DMSO.

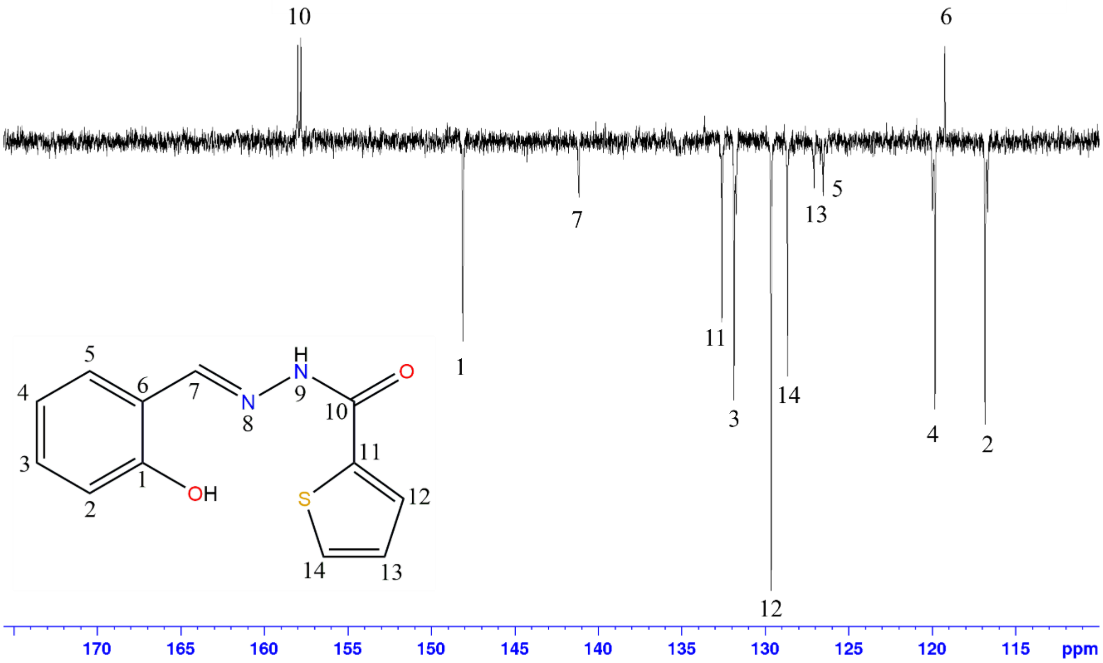

Figure S13. ^{13}C NMR spectra for the ligand H_2L in DMSO.

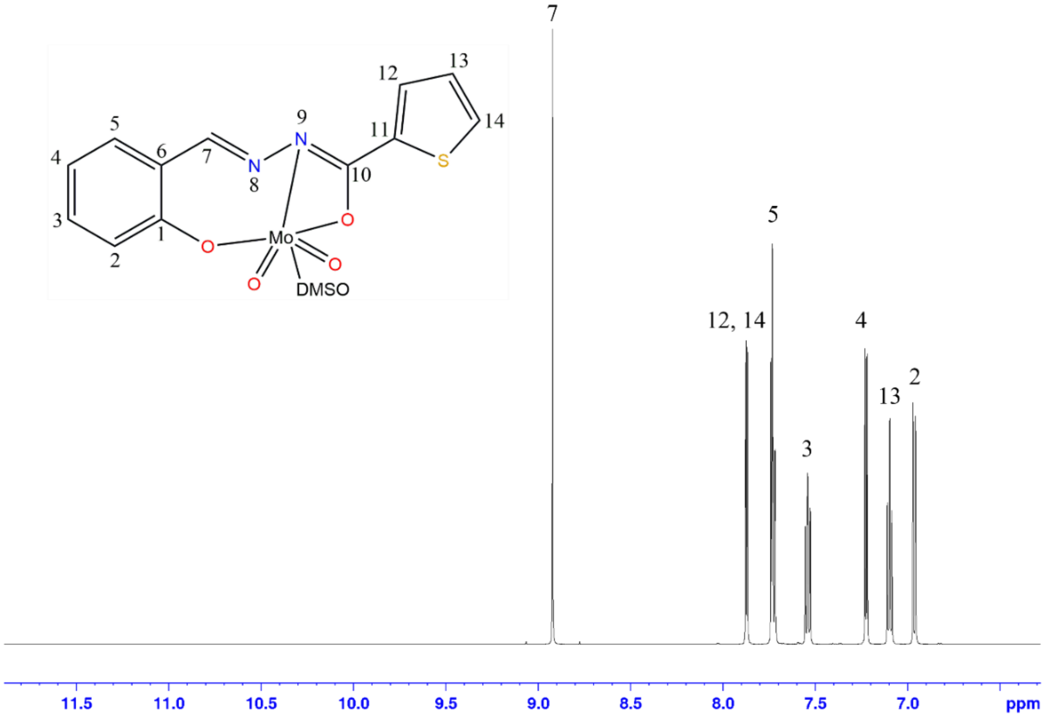

Figure S14. ^{1}H NMR spectra for the complex $[MoO_2(L)(MeOH)]$ in DMSO.

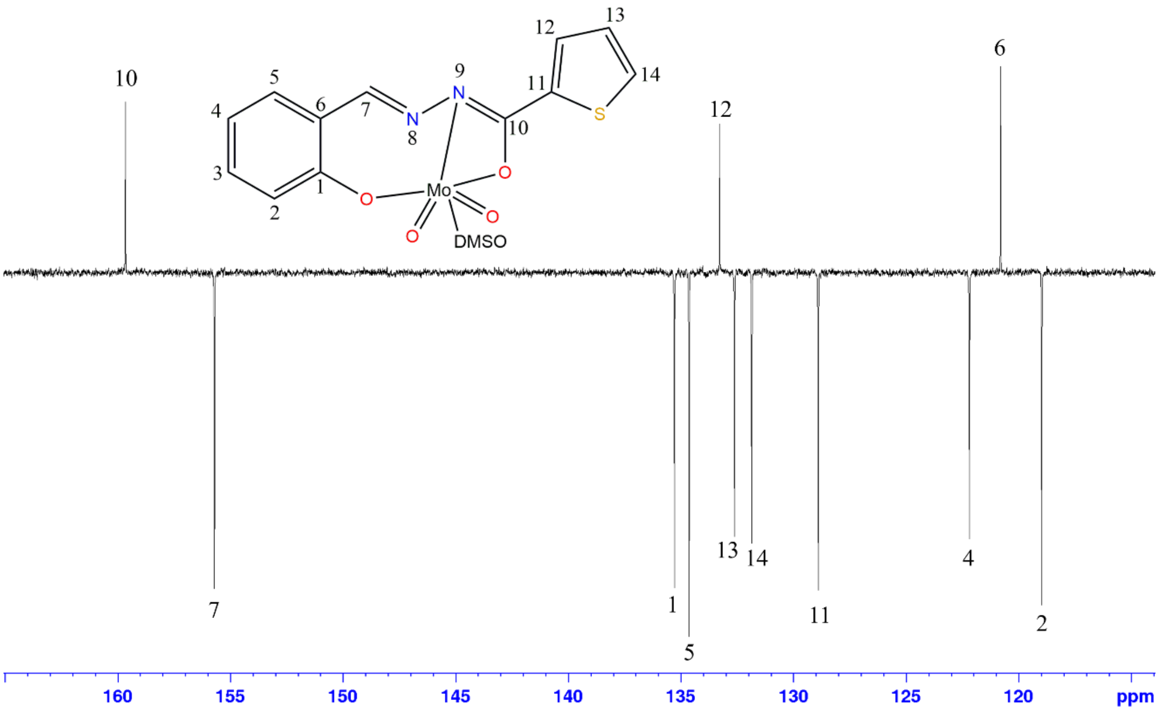

Figure S15. ^{13}C NMR spectra for the complex $[MoO_2(L)(MeOH)]$ in DMSO.
